# *Smart Pregnancy*: AI-Driven Approaches to Personalised Maternal and Foetal Health—A Scoping Review

**DOI:** 10.3390/jcm14196974

**Published:** 2025-10-01

**Authors:** Vera Correia, Teresa Mascarenhas, Miguel Mascarenhas

**Affiliations:** 1 Department of Obstetrics and Gynecology, Unidade Local Saúde Médio Ave, Largo Domingos Moreira, 4780-371 Famalicão, Portugal; 2 Faculty of Medicine, University of Porto, Alameda Professor Hernâni Monteiro, 4200-427 Porto, Portugal; tqc@sapo.pt (T.M.); miguelmascarenhassaraiva@gmail.com (M.M.); 3 Department of Obstetrics and Gynecology, São João University Hospital, Alameda Professor Hernâni Monteiro, 4200-427 Porto, Portugal; 4 Department of Gastroenterology, São João University Hospital, Alameda Professor Hernâni Monteiro, 4200-427 Porto, Portugal; 5 WGO Gastroenterology and Hepatology Training Center, 4200-427 Porto, Portugal; 6 CINTESIS@RISE, Department of Community Medicine, Information and Health Decision Sciences (MEDCIDS), Faculty of Medicine, University of Porto, 4200-427 Porto, Portugal

**Keywords:** artificial intelligence, machine learning, deep learning, obstetrics, maternal–foetal health

## Abstract

**Background/Objectives**: The integration of artificial intelligence (AI) into obstetric care poses significant potential to enhance clinical decision-making and optimize maternal and neonatal outcomes. Traditional prediction methods in maternal-foetal medicine often rely on subjective clinical judgment and limited statistical models, which may not fully capture complex patient data. By integrating computational innovation with mechanistic biology and rigorous clinical validation, AI can finally fulfil the promise of precision obstetrics by transforming pregnancy complications into a preventable, personalised continuum of care. This study aims to map the current landscape of AI applications across the continuous spectrum of maternal–foetal health, identify the types of models used, and compare clinical targets and performance, potential pitfalls, and strategies to translate innovation into clinical impact. **Methods:** A literature search of peer-reviewed studies that employ AI for prediction, diagnosis, or decision support in Obstetrics was conducted. AI algorithms were categorised by application area: foetal monitoring, prediction of preterm birth, prediction of pregnancy complications, and/or labour and delivery. **Results:** AI-driven models consistently demonstrate superior performance to traditional approaches. Nevertheless, their widespread clinical adoption is hindered by limited dataset diversity, “black-box” algorithms, and inconsistent reporting standards. **Conclusions:** AI holds transformative potential to improve maternal and neonatal outcomes through earlier diagnosis, personalised risk assessment, and automated monitoring. To fulfil this promise, the field must prioritize the creation of large, diverse, open-access datasets, mandate transparent, explainable model architectures, and establish robust ethical and regulatory frameworks. By addressing these challenges, AI can become an integral, equitable, and trustworthy component of Obstetric care worldwide.

## 1. Introduction

Artificial intelligence has become a foundational pillar of modern medicine, harnessing computational paradigms that parallel human reasoning to discern patterns, inform decisions and generate reliable predictions. Initially, symbolic systems encoded domain expertise directly as rules and ontologies; early medical expert systems translated clinical guidelines into sequences of if-then statements that guided diagnosis and treatment planning [1]. As clinical data volumes expanded, a migration toward data-driven methodologies gave rise to machine learning (ML), which enables algorithms to learn predictive relationships from empirical observations [1]. Supervised approaches such as logistic regression (LR) estimate the probability of clinical outcomes from patient variables, while decision trees (DT) partition the feature space into clinically meaningful strata [1]. Ensemble techniques, particularly random forests (RF) and gradient boosting machines exemplified by XGBoost, enhance predictive performance by combining numerous weak learners into robust consensus models [2]. Advances in computational power and algorithmic design piloted in the era of deep learning (DL), a subclass of machine learning characterized by multilayer neural networks that extract hierarchical representations directly from raw inputs [2]. Convolutional neural networks (CNN) analyse spatial hierarchies within medical images, empowering automated segmentation of anatomical structures and detection of subtle anomalies in modalities ranging from ultrasound to magnetic resonance imaging [2,3]. Recurrent neural networks (NN), including long short-term memory architectures, capture temporal dependencies within sequential data streams such as continuous foetal heart rate tracings or maternal physiological signals [3]. The introduction of transformer models, which employ self-attention mechanisms to contextualize every element of a sequence simultaneously, has revolutionized natural language processing and shows promise for modelling complex multimodal clinical data [4]. Complementary to predictive modelling, generative techniques including variational autoencoders and generative adversarial networks synthesize realistic data samples, thereby mitigating limitations imposed by scarce or imbalanced datasets [5]. As model complexity grows, interpretability emerges as a critical consideration. Tools such as SHapley Additive exPlanations (SHAP) and Local Interpretable Model-agnostic Explanations (LIME) decompose model predictions into contributions from individual features, thereby enhancing transparency and facilitating clinician trust [5].

Maternal-foetal medicine exemplifies a domain in which artificial intelligence’s full spectrum can be deployed to transform surveillance and intervention. Conventional prenatal monitoring relies on intermittent clinic visits, manual ultrasound calliper measurements and retrospective chart reviews, approaches that may fail to identify developing complications until they pose imminent risk. Contemporary machine-learning classifiers trained on extensive electronic health-record cohorts have demonstrated the ability to predict complications such as preterm birth several weeks in advance, with ensemble methods such as random forests and gradient boosting outperforming traditional regression models [6]. Deep-learning pipelines now automate foetal biometry with precision on par with expert sonographers, extracting head circumference and abdominal measurements directly from ultrasound video sequences [7,8]. Wearable sensor platforms integrate real-time signal processing algorithms to continuously monitor maternal vital signs and foetal movements, enabling personalised risk stratification and the delivery of timely clinical alerts [9].

The main goals of our research are threefold: First, to offer a comprehensive review of the state-of-the-art applications of artificial intelligence within prenatal and perinatal medicine; second, to critically examine the barriers to its widespread adoption, including data heterogeneity across populations, challenges in algorithmic interpretability, regulatory landscapes and ethical imperatives; and third, to uncover novel perspectives and highlight unresolved issues that could stimulate further investigation in this burgeoning area aimed at translating computational innovations into equitable and personalised maternal-foetal healthcare.

## 2. Materials and Methods

A scoping review was conducted in accordance with the Joanna Briggs Institute Methodology for JBI Scoping Reviews and adhering to the PRISMA Extension for Scoping Reviews (PRISMA-ScR) checklist [10,11].

### 2.1. Search Strategy and Selection Criteria

An approach encompassing dual methodology was applied: a semi-structured review that spotlights seminal works deemed to have significant impact, coupled with a systematic search to ensure a thorough and exhaustive synthesis of the current landscape. This integrative strategy allowed for provision of a robust, multifaceted analysis that not only maps the state-of-the-art in Obstetrics research but also illuminates avenues for future research.

A systematic search was conducted in May 2025 across scholarly databases including PubMed and WebofScience to identify relevant studies on AI applications in foetal medicine and Obstetrics. The search terms combined keywords for AI methodologies (“Artificial Intelligence”, “Machine Learning”, “Deep Learning”, “Neural Networks”, “CNN”, “DL”, “AI”, “ML”, “predictive model *”) with obstetric and fetal health descriptors (“Obstetric”, “Fetal medicine”, “Maternal-fetal medicine”, “Prenatal Diagnosis”, “Prenatal Screening”, “Fetal”, “Foetal”, “Maternal”, “Fetal Ultrasound”, “Obstetric Ultrasound”, “perinatal”, “Pregnancy”, “Noninvasive Prenatal Testing”, “Fetal Monitoring”, “Cardiotocography”, “CTG”, “Fetal Heart Rate”, “Fetal Hypoxia”, “Growth Restriction”, “Preterm”, “Preeclampsia”, “Gestational Diabetes”, “Postpartum Haemorrhage”, “Labor”, “Labour”, “Delivery”, “Birth”, “VBAC”, “Cesarean Section”, “Obstetric Surgery”).

### 2.2. Screening and Eligibility

Titles and abstracts were reviewed and conflicts resolved by discussion. Articles were excluded if they did not describe AI/ML/DL model development or evaluation in foetal or obstetric contexts, or if they were reviews, commentaries or abstracts. Full texts were assessed for inclusion based on predefined criteria: (i) original peer-reviewed research; (ii) application of AI/ML/DL for prediction, diagnostic support, decision-making or workflow optimization in maternal-foetal medicine/obstetrics; (iii) detailed methodological description and performance metrics; (iv) clinically applicable in foetal monitoring, prediction of preterm birth, prediction of pregnancy complications and/or labour and delivery.

### 2.3. Data Extraction

For each included study, we extracted: (a) year of publication; (b) type of study; (c) AI approach (algorithm type, training/validation strategy); (d) dataset characteristics (size, source, population); (e) performance measures (accuracy, sensitivity, specificity, AUC); (f) best-performing algorithm, if applicable; (g) limitations and (h) clinical applications. Discrepancies were reconciled by consensus.

### 2.4. Synthesis and Reporting

Data were synthesized narratively, grouping studies by clinical domain (e.g., AI for ultrasound imaging analysis, AI in foetal monitoring and risk prediction, AI-based prediction of preterm birth, AI in pregnancy complications, AI in labour and delivery) and AI technique. Emerging trends, methodological strengths and limitations were identified, and research gaps were highlighted to inform future directions in AI-enabled obstetric care.

## 3. AI Applications in Maternal-Foetal Medicine

**Figure 1:**
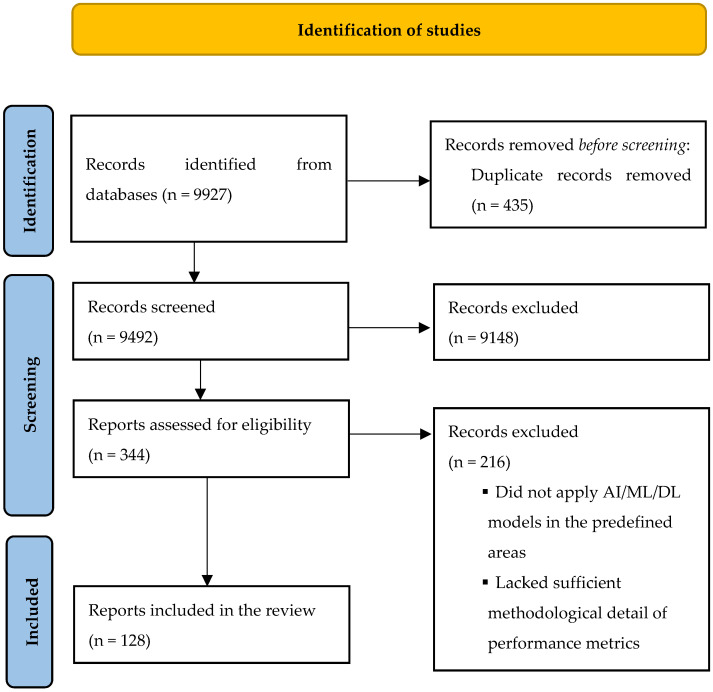
PRISMA flow diagram for identification of included studies.

The aforementioned search strategy produced 9927 records. After duplicate exclusion, 9492 unique records were screened, of which 344 met the criteria for full-text review. Of these, 128 were included in the review. Details of the study selection process are presented in Figure 1. Study characteristics are summarized in Table 1, Table 2, Table 3, Table 4, Table 5 and Table 6.

### 3.1. AI in Foetal Monitoring and Risk Prediction

Cardiotocography (CTG), introduced in the late 1960s, represented a pivotal advance in intrapartum foetal surveillance by enabling continuous recording of foetal heart rate (FHR) and uterine contractions (UC) to detect early signs of hypoxia [139,140]. However, more than fifty years on, definitive evidence that CTG reduces neonatal mortality or long-term neurological injury remains lacking, while its practical use is undermined by a high false-positive rate, poor specificity and substantial intra- and interobserver variability [139]. These shortcomings have contributed to rising caesarean delivery rates and missed opportunities for timely intervention, emphasising the need for more objective, reproducible analytic methods [139].

A transformative paradigm shift is now underway, moving from subjective waveform interpretation toward quantifiable, data-driven solutions that integrate advances in signal acquisition, engineering and AI. This transition necessitates international, multidisciplinary collaboration among clinicians, engineers, data scientists, device manufacturers and regulators, all aligned around well-defined, actionable perinatal outcomes.

The first computerized CTG systems of the 1980s automated extraction of classic features, including baseline rate, variability, accelerations and decelerations, to alert clinicians to abnormal tracings [140,141]. While these rule-based algorithms reduced some subjectivity, their reliance on small, retrospective datasets and expert-defined thresholds limited generalizability [140,141]. In the past decade, ML and DL methods have superseded these early efforts, incorporating advanced signal-processing techniques, such as phase-rectified signal averaging, wavelet transforms and time-frequency feature extraction, to uncover latent predictors of foetal compromise [142,143,144]. Hybrid models that combine CTG features with maternal and obstetric risk factors have achieved improved sensitivity and reduced false positives in retrospective cohorts, albeit prospective, external validation remains sparse [145,146].

Contemporary DL approaches have demonstrated significant gains in CTG interpretation. In one of the largest series to date, Park et al. trained an InceptionTime convolutional neural network on 124,777 intrapartum CTG recordings drawn from a nationwide, multicentre registry, achieving an area under the receiver-operating characteristic curve (AUC) of 0.89 for predicting severe neonatal acidemia (pH < 7.05) with 90% sensitivity at a positive-predictive-value threshold of 30%; external validation yielded an AUC of 0.72, highlighting the imperative for dataset-specific calibration [147]. Similarly, Ben M’Barek et al. developed DeepCTG^®^ 2.0, a CNN framework trained on 27,662 CTG tracings across three tertiary centres, which demonstrated AUCs of 0.74–0.83 for moderate to severe acidemia, outperforming its logistic-regression–based predecessor by approximately 0.05 in AUC [13].

Signal-quality correction has emerged as a critical preprocessing step. Boudet et al. employed a gated recurrent unit (GRU) network to distinguish true foetal from maternal heart rate and artefactual noise, achieving 93% sensitivity and a 96% positive predictive value, thereby strengthening inputs for downstream predictive models [31]. Notably, Frasch et al. demonstrated that DL applied to scanned analog CTG tracings could classify potentially preventable foetal distress with 94% accuracy, illustrating the utility of leveraging legacy datasets for large-scale model training [32]. More recently, Daydulo et al. combined Morse-wavelet time–frequency feature extraction with DL architectures to detect foetal distress, further validating the synergistic benefits of advanced signal processing and neural networks [29].

Accompanying the emerging Role of Generative Aim, the interpretative capacity of large language models (LLMs) has also been explored in this field. In a proof-of-concept comparison against junior and senior clinicians interpreting CTG screenshots, Gumilar et al. found that GPT-4o achieved a mean interpretive score of 77.9 (0–100 scale), closely approximating senior obstetricians (80.4) and outperforming less experienced practitioners, suggesting potential for generative AI–assisted decision support [14].

Despite these advances, clinical translation is impeded by several factors: (i) a predominantly retrospective evidence base, necessitating prospective, multicentre trials to establish safety and efficacy; (ii) heterogeneity in CTG acquisition protocols and annotation standards, underscoring the need for harmonized data standards; (iii) limited explainability of “black-box” models, which must be addressed through integrated visualization and interpretability tools; and (iv) nascent regulatory frameworks for utilization of AI/ML technology in Obstetrics.

Looking ahead, integration of AI-driven CTG analysis into electronic health records and central monitoring systems promises real-time decision support, automated risk stratification and standardised reporting. The formation of large, federated datasets governed by international standards will be essential to develop robust, generalizable models. Ultimately, by combining methodological rigor with collaborative, multidisciplinary implementation efforts, AI-enhanced CTG has the potential to transform intrapartum care, reducing perinatal morbidity and avoiding unnecessary interventions.

A summary of studies regarding AI applications in Foetal Monitoring is presented in Table 1.

### 3.2. AI-Based Prediction of Preterm Birth

Preterm birth (PTB), defined as delivery prior to 37 completed weeks of gestation, represents a critical global health challenge, accounting for approximately 10% of live births and remaining the foremost cause of neonatal morbidity and mortality worldwide [47]. Infants born preterm are predisposed to respiratory distress syndrome, intraventricular haemorrhage, necrotizing enterocolitis and long-term neurodevelopmental impairments, including cerebral palsy and cognitive delay, as well as chronic cardiovascular and metabolic sequelae extending into adulthood [148]. The aetiology of PTB is complex and multifactorial, encompassing spontaneous preterm labour precipitated by uterine overactivity or cervical insufficiency, infection-mediated inflammatory cascades, aberrant activation of the maternal–foetal hypothalamic–pituitary–adrenal axis, decidual haemorrhage and placental dysfunction, as well as medically indicated (iatrogenic) delivery for maternal or foetal compromise [148]. Established prophylactic strategies in women with prior PTB or asymptomatic transvaginal ultrasound short cervix, such as vaginal progesterone administration, cervical cerclage, pessary or a combination of these, have demonstrably reduced individual risk but remain suboptimal at the population level, in part because current clinical screening instruments identify only a subset of those destined to deliver prematurely [148]. Early and accurate stratification of PTB risk is therefore essential to optimize antenatal surveillance, deliver targeted therapies and allocate perinatal resources effectively.

Recent advances in AI have enabled the synthesis of high-dimensional data streams to improve PTB prediction beyond conventional risk factors. Kłoska and colleagues applied long short-term memory (LSTM), CNN and RF algorithms to combined uterine electromyography (EMG), tocodynamometry (TOCO) and electronic health record data from 1200 singleton pregnancies [47]. By extracting EMG features as burst frequency, interburst-interval variability and power spectral density in the 0.34–1.0 Hz band, and TOCO metrics, specifically contraction frequency and amplitude, and further integrating these with maternal age, body mass index, parity and prior PTB history, the LSTM model most effectively captured the temporal progression of uterine “activation,” achieving an area under the receiver-operating characteristic curve (AUC) of 0.87, a sensitivity of 0.83 and a specificity 0.85, significantly outperforming both CNN (AUC 0.83) and RF (AUC 0.79) [47]. In parallel, Ohtaka et al. trained a CNN on transvaginal cervical ultrasound video sequences (*n* = 59), quantifying dynamic changes in cervical funnelling and tissue echotexture to predict imminent PTB with an AUC of 0.92, a sensitivity of 0.88 and a specificity of 0.90, thereby demonstrating the feasibility of real-time, image-based risk stratification within routine obstetric practice [48].

Nonetheless, explainability and clinical interpretability are paramount for AI adoption. Clinicians must understand why an algorithm issues a high-risk alert before acting on it, verifying that predictions reflect known physiological mechanisms rather than spurious correlations. It is, therefore, relevant to denote work such as Kokkidinis and colleagues’, which combined extreme gradient boosting with SHapley Additive exPlanations (SHAP) on cervical length, foetal fibronectin, interleukin-6 and obstetric history (*n* = 500) to achieve an AUC of 0.89, sensitivity of 0.82 and specificity of 0.85, providing feature-level attributions that facilitate individualized patient counselling and shared decision-making [52]. Similarly, Andrade-Júnior et al. developed a stacked Bayesian extreme learning machine ensemble combining XGBoost, LR and neural class-labeling networks for iatrogenic PTB (*n* = 800), achieving an AUC of 0.91, sensitivity of 0.83 and specificity of 0.88, while preserving interpretability, illustrating how hybrid architectures can enhance robustness in heterogeneous clinical scenarios [51]. Together, these studies illustrate how explainable AI frameworks can bridge the gap between high-performance prediction and clinician trust, thereby accelerating adoption and ultimately improving perinatal outcomes.

By enabling identification of high-risk women weeks or even months before labour onset, these AI-driven models could materially reduce PTB incidence through timely administration of prophylactic interventions (e.g., progesterone, cerclage, pessary), optimised scheduling of antenatal corticosteroids and transfer to tertiary care centres. Moreover, precise risk stratification may prevent unnecessary interventions in low-risk women, thereby reducing healthcare costs and avoiding iatrogenic complications. However, current studies are constrained by retrospective, single-centre designs, limited sample sizes and the absence of external, prospective validation. Standardisation of signal acquisition protocols, harmonization of imaging techniques and rigorous assessment of cost-effectiveness and workflow integration will be critical in the years to come. Future research should prioritize large-scale, multi-institutional prospective trials, exploration of multimodal fusion including biochemical biomarkers and genomics, and development of clinician-centric interfaces to translate these high-performance algorithms into routine obstetric care and ultimately diminish the global burden of preterm birth.

A summary of studies regarding AI applications in prediction of preterm birth is presented in Table 2.

### 3.3. AI in Prediction of Pregnancy Complications

#### 3.3.1. AI for Early Prediction of Preeclampsia

Pre-eclampsia is among the oldest recognised complications of pregnancy, historically dubbed “the disease of theories” for the multitude of its proposed aetiologies [149]. Clinically defined by new-onset hypertension after 20 weeks’ gestation accompanied by end-organ involvement, it encompasses a spectrum from mild gestational hypertension with proteinuria to life-threatening syndromes, such as eclampsia, HELLP syndrome and maternal multiorgan dysfunction [150]. Maternal manifestations may emerge at any point antenatally or persist postpartum, and even ostensibly mild disease confers an elevated lifetime risk of cardiovascular and metabolic disorders for both mother and offspring [149,150,151].

Despite over a century of research implicating defective placentation as the central driver of early-onset pre-eclampsia, the precise interplay between placental pathology and maternal cardiovascular adaptation remains incompletely defined [149]. Normal pregnancy demands profound cardiovascular remodelling, namely plasma volume expansion, decreased systemic vascular resistance, and enhanced cardiac output; yet, in pre-eclampsia this adaptive capacity falters, precipitating hypertension, end-organ ischemia, and adverse perinatal outcomes [149]. These intertwined placental and maternal maladaptations continue to confound obstetrical care: screening algorithms rooted in clinical risk factors and standardised biomarkers achieve only modest predictive accuracy, and interventions such as low-dose aspirin yield benefit in only a subset of high-risk patients [150,151]. Pre-eclampsia risk stratification still relies primarily on clinical risk scores and simple multivariable regression models. The most widely implemented frameworks draw on maternal history (age, parity, pre-eclampsia in previous gestation), blood pressure, and basic biochemical markers, often transformed into multiples of the median (MoM), to generate population-level risk estimates. The *Fetal Medicine Foundation (FMF)* competing-risks model, for example, integrates maternal factors, mean arterial pressure (MAP), uterine artery pulsatility index (UtA-PI), placental growth factor (PlGF) and pregnancy-associated plasma protein-A (PAPP-A) in a Gaussian-based algorithm [152]. In large validation cohorts, this approach achieved AUCs of approximately 0.78 for any pre-eclampsia and 0.88 for preterm pre-eclampsia at a 10 % screen-positive rate [152]. Similarly, logistic-regression models that incorporate first-trimester uterine Dopplers and MoM-standardised biochemistry have reported detection rates of 55–60 % for all pre-eclampsia and 75–80 % for preterm cases at a 10 % false-positive rate [152]. While these models represent a step forward from univariate risk assessment, they remain limited by their reliance on linear combinations of preselected features, the need for MoM standardization, and suboptimal performance in heterogeneous populations. Thus, the imperative persists for novel strategies that can integrate multidimensional data, specifically clinical, hemodynamic, imaging and molecular, and reveal latent patterns predictive of disease onset and severity. In this context, AI offers a transformative paradigm, capable of transcending linear models to synthesize high-dimensional inputs and generate individualized risk trajectories that may guide truly precision-driven prevention, monitoring, and therapeutic intervention.

Recent AI-driven studies have pursued complementary strategies, such as deep integration of raw clinical and biochemical inputs, as well as large-scale EHR mining, collectively pushing beyond the plateau of aforementioned traditional methods.

Ansbacher-Feldman et al. bypassed MoM transformations by training a two-layer feed-forward neural network on 60,789 singleton pregnancies using raw maternal characteristics (age, BMI, parity, pre-eclampsia in prior gestation, race, type of conception, interpregnancy interval) and unstandardised biomarker concentrations (MAP, UtA-PI, PlGF, PAPP-A) [75]. At a 10 % screen-positive rate, their “posterior” model augmented with biomarkers increased detection of all pre-eclampsia cases from 41 % to 53 %, and of preterm pre-eclampsia from 53 % to 75 %, with AUC improvements from 0.77 to 0.82 (any PE) and from 0.82 to 0.91 (preterm PE) [81]. This direct ingestion of raw values exemplifies AI’s capacity to learn complex, nonlinear interactions that would be obscured by manual standardization.

In a population of 48,250 pregnancies drawn from a Southeast Melbourne health network, Tiruneh et al. demonstrated that “low-fidelity” EHR variables can yield robust predictions when ensembled in a random forest. Their model attained an AUC of 0.84 for pre-eclampsia versus controls, with similar discrimination for early and late-onset subtypes. Crucially, this approach leverages routinely captured, large-scale data without bespoke biomarker assays or specialized imaging, suggesting a path toward scalable risk stratification in diverse healthcare systems [75].

Zheng et al. combined semi-supervised U-Net segmentation of sagittal T2-weighted placental MRI with large-scale radiomic feature extraction—over 3000 wavelet, texture, and shape metrics—and fused these with logistic regression into a “deep learning radiomics” (DLR) signature. In a multicentre cohort of 420 pregnancies, the DLR model achieved AUCs of 0.84–0.89 for distinguishing pre-eclampsia from normotensive controls and 0.92 for combined PE plus foetal growth restriction. By quantifying subtle microstructural alterations in the placenta, radiomics reveals mechanistic links between villous architecture and disease risk [65].

Wang et al. interrogated GEO microarray datasets and a small validation cohort to identify 11 immune-related differentially expressed genes (DEGs), whose expression profiles were distilled by LASSO and random forest into a diagnostic panel. The resulting model achieved AUCs of 0.79 in test sets and 0.87 in external validation for early pre-eclampsia prediction. This “molecular radiology” of the maternal-foetal interface integrates immune–metabolic networks, offering both predictive and therapeutic insight [66].

Araújo et al. applied a LightGBM boosting model to routine complete blood counts in a Brazilian cohort, achieving an AUROC of 0.90 for severe pre-eclampsia detection, highlighting the diagnostic potential of inexpensive, widely available laboratory indices [77].

Zhou and colleagues used an Inception-ResNet-v2 convolutional network on retinal fundus images obtained before 20 weeks, achieving an AUC of 0.85 for pre-eclampsia prediction, and up to 0.88 when combined with clinical risk factors, underscoring the retinal microvasculature as a surrogate for systemic endothelial health [71].

AI models promise to redefine antenatal care by delivering individualized risk trajectories weeks to months before symptom onset, informing aspirin prophylaxis, intensified surveillance, and resource allocation. Where traditional FMF and logistic-regression models plateau around AUCs of 0.75–0.85, AI approaches routinely exceed these thresholds, often reaching 0.90–0.95 in well-curated cohorts. Yet, their translation remains constrained by retrospective study designs, “black-box” opacity, and the need for prospective, randomised evaluation of clinical impact. Data heterogeneity mandates standardised data curation and federated learning solutions. Finally, robust, explainable AI frameworks are essential to align predictive features with known pathophysiology and secure regulatory approval.

A summary of studies regarding AI applications in pre-eclampsia is presented in Table 3.

#### 3.3.2. AI-Driven Models for Gestational Diabetes Risk Stratification

Gestational diabetes mellitus (GDM) affects up to 15 % of pregnancies worldwide and is a major driver of both short- and long-term morbidity for mother and offspring [153]. Current screening paradigms are predicated on a 75 g oral glucose tolerance test (OGTT) performed at 24–28 weeks, identifying hyperglycaemia late in gestation, by which point maladaptive placental and foetal metabolic programming have often already been set in motion [153]. Such a reactive approach misses the opportunity to deploy targeted lifestyle or pharmacological interventions during the first and early second trimesters, when maternal insulin sensitivity and β-cell function evolve dynamically [154]. Major clinical guidelines share key shortcomings [155,156,157]. The Royal College of Obstetricians and Gynaecologists advocates early risk-factor assessment followed by selective OGTT at 24–28 weeks for women with persistent risk markers; the American College of Obstetricians and Gynecologists (ACOG) recommends universal OGTT at the same gestational window, with earlier testing for high-risk individuals; and Diabetes Canada similarly reserves OGTT for mid-pregnancy while endorsing early screening for those with obesity or prior GDM [155,156,157]. All three frameworks rely on binary glycaemic thresholds that fail to capture the continuum of risk and require fasting, multiple phlebotomies, and laboratory infrastructure that may be inaccessible in under-resourced settings [155,156,157,158].

ML and DL approaches can exploit routinely collected first-trimester data, such as electronic health-record variables, biochemical panels, anthropometric measures, ultrasound radiomics and even patient-reported lifestyle metrics, to generate continuous, individualized GDM risk scores long before conventional OGTT. By stratifying risk in early pregnancy, AI-driven tools have the potential to triage women to intensified surveillance or preventive therapy, conserve resources by deferring OGTT for low-risk individuals, and tailor interventions (nutritional counselling, exercise programmes or metformin initiation) to those most likely to benefit from them.

Recent studies illustrate the promise of this paradigm. Broadly, these efforts can be grouped into three methodological categories: (i) structured-data models built on electronic health records (EHRs) and laboratory values; (ii) image-based radiomics and deep-learning pipelines; and (iii) hybrid or dual-task frameworks that combine clinical, biochemical and imaging inputs.

Several large retrospective cohorts harnessed routinely collected demographic and biochemical data to train ensemble learners. Hu X et al. leveraged 20 first-trimester EHR variables, including prior GDM history, HbA_1_c, mean arterial pressure and lipid panels, to achieve an AUC of 0.946 using XGBoost, dramatically outperforming logistic regression (AUC 0.946 AUC 0.752) [96]. Similarly, Zhao et al. applied NearMiss resampling to address class imbalance in a 103,172-pregnancy database, with a multilayer perceptron reaching AUC 0.943 versus 0.777 for multivariate logistic regression [90]. These studies illustrate that complex, nonlinear ensembles can extract subtle interactions among clinical predictors that linear models miss, and that careful data-preprocessing (e.g., resampling) is critical when GDM prevalence is low.

By contrast, Bigdeli et al. demonstrated the challenges of dual-task modelling: in a single-centre Iranian cohort, a random-forest algorithm predicted OGTT positivity with high fidelity (AUC 0.94) but struggled to forecast subsequent insulin requirement (AUC 0.64), highlighting how model performance can vary widely depending on endpoint prevalence and data completeness [89]. Kadambi et al. and Liao et al. further underscore the trade-offs of including specialized behavioural and self-monitored glucose metrics: while super-learner ensembles attained C-statistics up to 0.934 in discovery, simplified logistic models still achieved respectable AUCs (~0.80) with far greater interpretability [98,104].

Zhou et al. and similar radiomics studies pioneered the use of first-trimester ultrasound texture features to predict GDM [92]. By extracting >1300 quantitative placental features and combining them with deep-learning convolutional neural network (CNN) scores, the authors produced a nomogram with AUCs of 0.93 (discovery) and 0.88 (validation) [92]. This paradigm offers an elegant “no-blood-draw” risk stratification, seamlessly integrating into routine nuchal translucency scans. Nevertheless, manual region-of-interest delineation and ultrasound protocol variability present barriers to scalability, and external validation in larger, multi-ethnic cohorts remains pending [92].

Preconception and mobile-health tools, such as those developed by Kumar et al. in the S-PRESTO and GUSTO cohorts, illustrate how web-based interfaces can empower women to input lifestyle, anthropometric and basic laboratory data [101,102]. CatBoost and stacked-ensemble pipelines achieved AUCs of 0.82–0.83, demonstrating that patient-reported inputs combined with minimal biochemistry can approximate the performance of EHR-driven models [101,102]. Such platforms hold promise for low-resource settings, though their reliance on self-report introduces potential biases.

These models can be seamlessly integrated into obstetric electronic medical-record systems to flag high-risk women at booking visits, prompting clinicians to initiate dietary and exercise interventions. Ultrasound-augmented platforms can deliver automated GDM risk estimates during routine first trimester scans without additional patient burden. Web and smartphone-based preconception tools enable women to check personalised GDM risk based on self-reported data and first-trimester labs, empowering proactive lifestyle changes even before conception.

Despite impressive retrospective metrics, the majority of published models are single-centre and lack external validation across diverse ethnic and socioeconomic populations. Small sample sizes, extensive data exclusions, and reliance on high-dimensional biomarker panels limit generalizability to low-resource settings. Heterogeneous variable definitions and missing data in electronic health records threaten model robustness; concomitantly, “black-box” architectures without transparent feature attribution risk undermining clinician trust.

Artificial intelligence offers a transformative avenue to shift gestational diabetes care from a reactive, mid-pregnancy diagnostic model to a proactive, first-trimester precision-medicine approach. By harnessing diverse clinical, biochemical, imaging, and behavioural data, ML and DL models can identify at-risk women early, guide personalised interventions, and optimise resource allocation, ultimately improving maternal and neonatal health outcomes on a global scale. To better achieve the promise of AI in GDM care, large-scale, prospective, multi-centre trials are imperative to validate and calibrate risk models across populations and healthcare systems. Embedding explainability frameworks such as SHAP or LIME can illuminate key predictors and facilitate clinician acceptance. Health-economic analyses should compare the cost-effectiveness of AI-guided early interventions against standard OGTT-driven protocols. Finally, alignment of continuous AI-derived risk scores with established guideline thresholds will be essential to integrate these tools into existing care pathways.

A summary of studies regarding AI applications in GDM is presented in Table 4.

#### 3.3.3. AI in Predicting Postpartum Haemorrhage

Postpartum haemorrhage (PPH), conventionally defined as blood loss ≥ 500 mL within 24 h of delivery, remains the leading cause of maternal mortality worldwide, accounting for over 20% of maternal deaths and disproportionately affecting low and middle-income countries, where more than 90% of these deaths occur [159]. In high-resource settings, PPH still contributes to 8–19% of maternal deaths and drives substantial transfusion requirements and morbidity [160]. Early identification of women at elevated risk enables implementation of evidence-based prophylaxis, such as active management of the third stage of labour, timely administration of uterotonics, tranexamic acid, and pre-positioning of blood products [161].

Traditional risk-assessment tools rely on summative clinical scores that assign points for factors including previous PPH, multiple gestation, pre-eclampsia and prolonged labour [162]. Although readily deployable at the bedside of the patient, these instruments demonstrate only modest discrimination and often lack sensitivity for high-risk individuals; for example, a widely cited clinical score achieved an AUROC of 0.68 (95% CI 0.63–0.72) in validation cohorts [162]. Despite being simple to implement, these scores typically achieve only modest discrimination and do not account for complex, nonlinear interactions among maternal, labour, and facility variables [162]. Moreover, estimates of blood loss based on visual inspection remain notoriously inaccurate, further limiting timely recognition and management.

Recent advances in artificial intelligence, notably ML and DL algorithms, offer the capacity to integrate hundreds of peri-partum variables and to model intricate relationships that defy traditional regression. Ahmadzia and colleagues applied gradient-boosting, random forest, support-vector machine and multilayer perceptron models to the Consortium on Safe Labor dataset (*n* = 228,438), achieving an AUROC of 0.833 and a precision–recall AUC of 0.210 for a composite transfusion-PPH endpoint; the gradient-boosting model’s top predictors included mode of delivery, incremental oxytocin dose, tocolytic use, presence of an anaesthesia nurse and hospital care level [108].

Wang et al. evaluated five algorithmic approaches in 6144 caesarean deliveries, finding that a random forest model minimized prediction error of actual blood loss using a combination of 27 antepartum and intrapartum laboratory and clinical features, achieving a mean absolute error 21.7 mL (< 5.4% error) and root-mean-squared error 33.75 mL [109]. In a low-resource setting, Holcroft et al. developed a random-forest model in a Rwandan case–control cohort (*n* = 430) that predicted PPH on admission with 80.7% sensitivity and 71.3% specificity, relying solely on nine readily available variables including haemoglobin, maternal age, insurance status and obstetric history [110].

Albeit without external validation, the power of ensemble methods is further exemplified by Westcott et al., who trained gradient-boosted decision trees on 497 EHR features in 30,867 US deliveries to reach an AUROC of 0.979 and an overall accuracy of 98.1% [111]. Earlier work by Akazawa et al. (*n* = 9894) and Venkatesh et al. (*n* ≈ 152,000) demonstrated reproducible performance of XGBoost and random-forest models with C-statistics around 0.93 in large multicentre cohorts, while highlighting challenges of missing data and dated predictor sets [113,114].

These AI-driven models can be seamlessly embedded within electronic medical record systems to provide real-time risk scores at labour admission or preoperative evaluation, triggering tailored prophylaxis and facilitating resource allocation. In low-resource contexts, simplified calculators derived from more complex models can guide interventions when blood-bank capacity is limited. Furthermore, AI-based estimators of actual blood loss may improve quantification, enabling earlier transfusion and reducing maternal morbidity.

Despite impressive retrospective performance, most published models suffer from single-centre derivation, small or selective sample sizes, heterogeneous variable definitions, and limited external validation, raising concerns about overfitting and generalizability. The “black-box” nature of many ML/DL algorithms further hampers clinical trust in the absence of transparent explainability frameworks.

Future research should prioritize prospective, multicentre validation studies across diverse healthcare settings; incorporation of explainable-AI techniques (e.g., SHAP, LIME) to elucidate key predictors; alignment of model thresholds with clinical prophylaxis protocols; and health-economic assessments to determine cost-effectiveness. By addressing these challenges, AI has the potential to transform PPH management from reactive treatment to proactive prevention, thereby reducing the global burden of maternal haemorrhage.

A summary of studies regarding AI applications in PPH is presented in Table 5.

### 3.4. AI Applications in Labour and Delivery

Accurate prediction of delivery mode is essential for personalised intrapartum management, optimal resource allocation and reduction in unnecessary surgical interventions. Traditional risk stratification relies on a limited set of static clinical variables, which have demonstrated only modest discriminatory power.

Recent advances in AI have enabled the integration of dynamic intrapartum signals, comprehensive electronic health record data and ultrasound biometry to achieve substantially higher predictive accuracy. Ricciardi et al. demonstrated that a Random Forest classifier trained on 17 CTG-derived features (including foetal heart rate variability, spectral power and Poincaré metrics) could predict the need for caesarean delivery with 91.1% accuracy and an AUC of 0.967 [135]. In practice, this model could be integrated into monitoring systems to alert clinicians when CTG patterns portend operative delivery, prompting earlier obstetric review and preparation of surgical teams [135]. Similarly, Fergus et al. showed that an ensemble of Fisher’s linear discriminant analysis, RF and SVM achieved an AUC of 0.96 on 552 intrapartum CTG tracings [137]. Clinically, such an ensemble could serve as a second-opinion tool in labour wards to reduce inter-observer variability in CTG interpretation, support decisions about assisted vaginal delivery versus caesarean section and potentially avert adverse outcomes from delayed interventions.

Islam et al. applied a Henry Gas Solubility Optimization–enhanced RF to >20,000 births from demographic and health surveys, attaining nearly 98.3% accuracy in caesarean section prediction [129]. Although derived from survey data, this model could inform public health planning by identifying geographic or demographic groups at elevated risk of caesarean delivery, thereby guiding targeted training, resource deployment and policy interventions in low-resource settings [129].

Meyer et al. developed an XGBoost model on 73,667 deliveries at a tertiary centre, incorporating cervical dilation, ultrasound-adjusted foetal biometry and labour progression metrics. Deployed as a web-based calculator, it enables clinicians to provide individualized caesarean counselling on admission, sharing quantified risk estimates with patients to support shared decision-making and consent processes [126].

Zhang et al. trained a Random Forest on 2552 caesarean cases using detailed electronic medical record variables such as maternal demographics and ultrasound parameters, achieving an AUC of 0.979. This high-precision model could be embedded in electronic health systems to flag patients most likely to require caesarean delivery, streamlining anaesthetic planning, operating-room scheduling and blood-bank readiness [124].

Similarly, Lodi et al. applied a Probability Forest to predict caesarean delivery in 410 class III obese nulliparas (AUC 0.70). Although performance was lower, this targeted tool offers the first risk calculator for a notoriously challenging subgroup, allowing obstetricians to provide tailored counselling on delivery mode, anticipate operative difficulties and mobilize additional support for high-risk patients [123].

Regarding accurate prediction of labour induction success, which is critical to minimize unnecessary interventions, avoid prolonged hospital stays and reduce the risk of emergency caesarean delivery, Tingting Hu and colleagues retrospectively developed and validated a suite of machine-learning models, including logistic regression, naïve Bayes, support vector machine and AdaBoost, on 907 term pregnancies undergoing oxytocin induction (495 primiparous women; 312 multiparous women) [127]. Their logistic regression model achieved an AUC of 0.84 for primiparous and 0.89 for multiparous women, with external validation success rates of 94.2% and 96.6%, respectively, demonstrating robust discrimination based on clinical and sonographic variables such as Bishop score, foetal weight and amniotic fluid index [127]. In a multinational secondary analysis of two phase-III randomised trials (*n* = 1107), D’Souza et al. applied an unspecified machine-learning algorithm to predict successful induction in women with low Bishop scores (<4), yielding an AUC of 0.73 and identifying parity, gestational age and maternal BMI as leading predictors [125]. More recently, Liu et al. incorporated transvaginal ultrasound-derived cervical maturity features into XGBoost, CatBoost and random forest models in 101 women, with XGBoost achieving a mean absolute error of 13.49 h and RMSE of 16.98 h for prediction of induction-to-delivery interval, significantly outperforming the traditional Bishop score (MAE 19.45 h; RMSE 24.55 h) [122].

In addition to predicting primary caesarean delivery, AI has been applied to the challenge of vaginal birth after caesarean (VBAC). VBAC offers significant benefits over repeat caesarean delivery, including reduced surgical morbidity and faster maternal recovery, yet carries risks such as higher rates of uterine rupture and need for emergency intervention when compared to an elective caesarean section. Historically, VBAC candidacy has been guided by tools such as the Grobman calculator, developed by the Eunice Kennedy Shriver National Institute of Child Health and Human Development Maternal–Fetal Medicine Units (MFMU) Network, which estimates the probability of successful trial of labour after caesarean (TOLAC) using six readily available admission variables: maternal age, body mass index, race/ethnicity, history of prior vaginal delivery, indication for the previous caesarean and timing of that surgery [163]. Although externally validated across diverse populations, the Grobman model exhibits modest discrimination (AUC-PR 0.325 ± 0.067), limiting its precision for individualized counselling [163].

AI–driven approaches have sought to enhance VBAC prediction by incorporating high-dimensional clinical and real-time labour data. Macones et al. compared a back-propagation neural network with multivariate logistic regression in a case–control cohort of 400 women (100 failed TOLAC, 300 successful VBAC) and found that logistic regression (sensitivity 77%, specificity 65%, accuracy 69%) outperformed the neural network (sensitivity 59–63%, specificity 42–44%) in discriminating VBAC success [164]. More recently, Meyer et al. developed and externally validated Random Forest, XGBoost and generalized linear models on 989 consecutive TOLAC deliveries at a tertiary academic centre. The Random Forest achieved the highest area under the precision–recall curve (AUC-PR 0.351 ± 0.028), compared with XGBoost (0.350 ± 0.028) and GLM (0.336 ± 0.024), surpassing the traditional MFMU-Calculator prediction model (0.325 ± 0.067) [134]. Key predictors included prior vaginal birth, maternal height and arrest of descent. Clinically, these models could be integrated into decision-support tools to provide point-of-care VBAC success probabilities, informing counselling, consent and labour management in women considering TOLAC.

With respect to accurate identification of women at elevated risk for mediolateral episiotomy during the second stage of labour, it is well-known that it remains elusive, and its indiscriminate application can increase maternal morbidity while under-use may predispose to severe perineal trauma [116]. Tingting Hu and colleagues prospectively evaluated multiple AI algorithms, including support vector machine, random forest, LightGBM and XGBoost, on 1191 vaginal deliveries (incorporating 300 episiotomies) and demonstrated that the SVM model achieved the highest discriminative performance (AUC 0.882; recall 0.981; precision 0.790) [116]. Key predictors encompassed maternal characteristics (age, body mass index, parity), perineal metrics (length, elasticity, thickness, oedema), labour dynamics (duration of each stage, uterine contraction patterns) and intrapartum complications (shoulder dystocia, instrumental assistance) [116]. Embedding such a model into electronic monitoring systems or mobile decision-support tools could provide clinicians with real-time risk estimates at the bedside, enabling targeted episiotomy only for those most likely to benefit, thereby minimizing unnecessary perineal injury and optimising maternal outcomes.

Despite these promising results, most studies remain retrospective and single-centre, with potential selection bias, limited external validation and variability in data acquisition protocols. Intrapartum signal-based models require standardised cardiotocography sampling and preprocessing, while electronic health record–driven algorithms depend on data completeness and consistent coding practices. Future research must prioritize prospective, multicentre trials to confirm generalizability, incorporate continuous ultrasound and wearable sensor streams for richer temporal modelling, and adopt explainable AI frameworks that provide transparent feature attributions. Seamless integration into clinical workflows, including real-time inference engines and user-centric interfaces, will be essential to translate these high-performance models into routine obstetric practice and ultimately improve maternal and neonatal outcomes.

A summary of studies regarding AI applications in labour and delivery is presented in Table 6.

## 4. Challenges and Limitations: Ethical and Regulatory Frameworks for AI in Obstetrics

The rapid adoption of AI in the continuum of Obstetric care promises substantial improvements in outcomes but also raises complex legal and ethical challenges. To ensure that AI deployments respect patient autonomy, privacy, and equity, and that they meet rigorous safety standards, stakeholders must navigate a multifaceted regulatory landscape while embedding robust ethical safeguards into every stage of development and implementation.

### 4.1. Current AI Legal Frameworks in Healthcare

International organizations and national governments are rapidly shaping the legal landscape to govern AI applications in healthcare. The World Health Organization (WHO) has issued comprehensive digital-health guidelines that articulate ethical principles for AI in medicine, emphasising respect for patient autonomy, equity of access, privacy protection, and ongoing evaluation of safety and effectiveness [165]. In 2024, the Organization for Economic Co-operation and Development (OECD) released its updated intergovernmental standard on trustworthy AI, calling for AI systems that are human-centered, fair, transparent, robust, and environmentally sustainable [166]. These high-level frameworks provide foundational guidance but lack binding legal force, leaving national regulators to translate principles into enforceable rules.

In the United States of America, the Health Insurance Portability and Accountability Act (HIPAA) Privacy Rule requires covered entities and business associates to implement administrative, physical, and technical safeguards, such as de-identification, encryption, and access controls to protect electronic protected health information (ePHI) used in AI model development and deployment [167]. Concurrently, the Food and Drug Administration (FDA) treats clinical AI/ML algorithms as Software as a Medical Device (SaMD). Its 2019 Discussion Paper and 2021 AI/ML-Based SaMD Action Plan establish a Total Product Life-Cycle approach, mandating pre-specified Change Control Plans for continuous-learning models, adherence to Good Machine Learning Practices (GMLPs), and post-market surveillance to monitor for performance drift and emerging risks [168,169].

The EU’s General Data Protection Regulation (GDPR) extends stringent protections to any processing of “personal data,” including health information [170]. AI projects must comply with GDPR’s principles of lawfulness, transparency, data minimization, and purpose limitation, obtain explicit consent or invoke a research exemption for “special category” data, and conduct Data Protection Impact Assessments for high-risk processing [168]. Building on GDPR, the upcoming AI Act (effective August 2026) introduces a risk-based classification: “high-risk” AI must satisfy rigorous requirements for data quality, human oversight, transparency, and conformity assessment under the Medical Device Regulation (MDR 2017/745) [171].

### 4.2. Clinical Investigation and AI-Specific Requirements

The translation of AI algorithms from prototype to clinical utility in Obstetrics necessitates rigorous investigation akin to that required for medical devices and pharmaceuticals. Regulatory bodies in major jurisdictions have delineated pathways to ensure that AI systems demonstrate safety, effectiveness, and generalizability before—and after—deployment.

In the United States, AI tools intended to inform clinical decision-making fall under the FDA’s definition of Software as a Medical Device (SaMD) [172]. When algorithm outputs will directly influence patient management, sponsors must secure an Investigational Device Exemption (IDE) before initiating clinical studies [172]. IDE applications must include a detailed protocol describing: the intended use and clinical role of the AI system; the characteristics of the training and validation datasets (including demographic composition and handling of missing data); performance–metric thresholds that would trigger study modification or termination; and data-safety monitoring procedures to detect adverse events or model failures in real time [172].

Under the European Medical Device Regulation (MDR 2017/745), AI/ML-based SaMD classified as “high-risk” must undergo clinical performance studies in accordance with Annex XIV. Such studies require ethics committee approval and, for devices in risk class IIa or higher, a formal Clinical Investigation Plan submitted to a Notified Body. The investigation plan must specify objectives, study design, sample-size justification, inclusion of appropriate comparator arms (for example, standard-of-care risk scores), and statistical analysis methods to evaluate primary endpoints such as sensitivity, specificity, and calibration in target populations [173].

To promote reproducibility and facilitate regulatory review, professional guidelines have been extended to AI interventions. The SPIRIT-AI extension provides 15 additional items for trial protocols, covering aspects such as algorithm versioning, data provenance, and human-in-the-loop mechanisms [174]. Similarly, CONSORT-AI defines 14 extension items for trial reporting, including clear description of integration into clinical workflows, criteria for expert override, and post-hoc explainability analyses to contextualize performance in subgroups (for example, across different ethnicities or gestational ages) [175].

Given that many AI algorithms incorporate adaptive or continuous-learning components, regulators require ongoing post-market monitoring to detect performance drift when real-world patient characteristics or care processes diverge from the development environment.

## 5. Conclusions and Future Directions

This scoping review provides a comprehensive synthesis of current AI applications across the maternal–foetal continuum. By capturing the methodological diversity of AI approaches while emphasising clinical relevance, it highlights both the transformative potential of AI for early diagnosis, personalised risk stratification, and automated monitoring and the emerging importance of explainable AI, multimodal data integration, and regulatory oversight.

However, several limitations warrant consideration. As a scoping review, no formal assessment of study quality or risk of bias was undertaken, limiting the ability to weigh the relative strength of evidence. The restriction to English-language publications may have introduced language bias, and the inclusion of studies spanning more than two decades introduces heterogeneity in data sources, model architectures, and reporting standards. Moreover, the predominance of retrospective, single-centre studies with limited external validation constrains generalizability.

To fully realize AI’s promise in obstetrics, several challenges and gaps must be addressed. Firstly, improving AI training datasets is crucial. Many current models have been developed on relatively narrow datasets, which can introduce algorithmic bias and limit their generalizability. There is an urgent need for larger and more diverse maternal health data sources to ensure that AI tools perform well across different populations and care settings. Encouraging open access to maternal-foetal datasets and establishing data-sharing collaborations will be instrumental in this effort, as it allows researchers to validate and refine AI models on a wide range of real-world scenarios. By closing the data gap and enhancing data quality, we can increase the fairness, accuracy, and clinical reliability of AI predictions in obstetrics.

Secondly, strengthening the collaboration between AI researchers and healthcare professionals is essential. Interdisciplinary teamwork can ensure that the next generation of AI tools is both clinically relevant and user-friendly for those on the front lines of care. Obstetricians, midwives, and maternal-foetal medicine specialists should be actively involved in the design, testing, and implementation of AI systems, providing valuable insights into clinical workflows and decision-making nuances. Likewise, computer scientists and engineers can tailor their algorithms to address the real-world needs and constraints identified by healthcare providers. In tandem, medical education will need to evolve to build AI literacy among clinicians. Training programs and continuing education could incorporate basic data science and AI concepts so that obstetrical care providers feel comfortable interpreting AI outputs and maintaining appropriate oversight of AI-assisted decisions. Fostering this two-way exchange, where technology experts and clinicians learn from each other, will help ensure trust in AI integration.

Thirdly, robust regulatory frameworks and ethical guidelines are needed to support safe AI deployment in Obstetrics. Given the high stakes in maternal-foetal medicine, it is imperative that AI algorithms undergo rigorous evaluation and approval processes before being widely adopted. Regulatory bodies and professional organizations should establish clear standards for validating obstetric AI tools, including requirements for transparency and thorough testing. Issues such as data privacy, informed consent for AI use, and liability in the event of AI mistakes also need defined policies. Importantly, ongoing oversight is required even after deployment: models may drift in performance over time or behave unpredictably in new settings, so continuous monitoring and periodic re-certification of AI systems should be part of the governance framework.

In summary, albeit its current nascent nature in clinical practice, artificial intelligence offers a transformative opportunity to improve obstetric outcomes and drive more personalised, equitable care. With continued interdisciplinary research, responsible deployment, and robust oversight, AI can be harnessed to help make pregnancy and childbirth safer, ushering in a new era of “intelligent” obstetric care that complements and strengthens the work of healthcare professionals.

## Figures and Tables

**Table 1:** AI applications in cardiotocography.

Reference	Country	Data Source	Best-Performing AI Model	Performance Metrics	Clinical Utility
**McCoy et al. (2025)** [12]	USA	Internal: 124,777 CTGs; External: 552 CTGs	IncTime architecture	AUC: 0.85 (pH < 7.05); 0.89 (pH < 7.05 + base excess < −10); Sens: 90%, Spec: 48% (for PPV 30%)	DL system for intrapartum detection of foetal acidemia
**M’Barek et al. (2025)** [13]	France	27,662 CTGs	CNN with pretraining and combined FHR + UC inputs	AUC (Severe acidemia): 0.74–0.83. AUC (Moderate + Severe): 0.70–0.83. Improved vs. DeepCTG 1.0 by ~0.05 AUC	Improved detection of neonatal acidemia compared to traditional and earlier DL models
**Gumilar et al. (2025)** [14]	Indonesia	7 CTGs	GPT-4o	Mean performance scores (0–100 scale): SHDs: 80.43, GPT-4o: 77.86, Gemini: 57.14, Copilot: 47.29; CG4o surpassed others.	Promising tool for aiding less experienced clinicians in CTG interpretation.
**Roozbeh et al. (2025)** [15]	Iran	7166 deliveries	RF	RF: AUC 0.77, Acc 0.77, Prec 0.72	Supports early identification of NFH risk using routine clinical data.
**Zhao et al. (2024)** [16]	China	552 CTGs	SE-TCN with CMFF (MHA)	Acc: 96.8%; Sens: 96.0%; Spec: 97.5%; Prec: 97.5%; F1-Score: 96.7%	Automates foetal acidosis diagnosis.
**Tarvonen et al. (2024)** [17]	Finland	4988 CTGs	SALKA	Cohen’s Kappa: 0.981; Sens: 0.981; PPV: 0.822; False-negative rate: 0.01	Enables automated, real-time HRV detection comparable to experts, especially in neonatal acidemia cases.
**Mushtaq et al. (2024)** [18]	India	2126 CTGs	DNN	Acc 0.99; Sens 0.93; Spec 0.93; AUC 0.96, Precision 0.93	High-performance, interpretable tool for CTG classification.
**Melaet et al. (2024)** [19]	Netherlands	678 CTGs (train *n* = 548; validation *n* = 87)	Patient-specific FHR predictor NN	AUC 0.96 for distinguishing normal vs. pathological segments	Enables earlier prediction of foetal compromise.
**Wahbah et al. (2024)** [20]	Japan and USA	70 pregnant women	BiLSTM-based DL framework with signal enhancement techniques	Subject-dependent accuracy: 94.2%, F1-score: 0.97; Subject-independent accuracy: 88.8%, F1-score: 0.96	Enables accurate noninvasive foetal ECG extraction and foetal heart rate estimation.
**Mendis et al. (2024)** [21]	Australia	552 CTGs	FHR-LINet	25% reduction in the time taken to detect foetal compromise compared to the state-of-the-art multimodal CNN	Enables earlier prediction of foetal compromise
**Li J et al. (2024)** [22]	China	Source domain:16,355 CTGs; Target domain: 3351 CTG	DSSDA-MMEDI (GoogLeNet with MME, DI, and DGMI integration)	Acc: 80.14%, Sens: 74.52%, Spec: 83.22%, F1-score: 72.67%, Kappa: 57.08%, MCC: 57.13%, AUC: 0.8502	Enables earlier prediction of foetal compromise.
**Das et al. (2023)** [23]	Bangladesh	125 CTGs	MLP with fuzzy annotations	Acc: 97.94%, ROC: 0.999	Accurately identifies Early, Late, and Variable decelerations.
**Liang et al. (2023)** [24]	China	552 CTGs, enhanced to 4738 samples	1D-CNN + BiGRU hybrid	Acc: 95.15%, Sens: 96.20%, Spec: 94.09%, F1-score: 95.20%, AUC: 99.29%	Real-time CTG classification and hypoxia risk detection
**Zhou Z et al. (2023)** [25]	China	552 CTGs	TGLCN	Acc: 89.80%	Improves classification accuracy and interpretability of CTG.
**Lee KS et al. (2023)** [26]	South Korea	5249 CTGs, 141,001 5-min samples	2D ResNet CNN	Sens: 98.0%, Spec: 99.5%, F1-score: 98.7%	Enables real-time foetal health classification via mobile app and server; supports remote antepartum monitoring
**M’Barek I et al. (2023)** [27]	France	1527 CTGs	LR	AUC: CTU-UHB (0.743), Beaujon (0.739), SPaM (0.768–0.873); improved specificity (12% FPR vs. 25% obstetricians)	Enables earlier prediction of foetal compromise.
**Cao Z et al. (2023)** [28]	China	16,355 CTGs	CNN for CTG + LGBM for classification using multimodal features	Acc: 90.77%, AUC: 0.9201, Normal-F1: 0.9376, Abnormal-F1: 0.8223, Prec: 82.83%, Spec: 93.15%	Supports early and intelligent antepartum screening.
**Daydulo et al. (2022)** [29]	Ethiopia	552 CTGs	ResNet-50 with Morse wavelet transform	1st stage labour: Acc 98.7%, Sens 97.0%, Spec 100% 2nd stage labour: Acc 96.1%, Sens 94.1%, Spec 97.7%	Reliable, automated FHR analysis for both early and late labour stages.
**Spairani et al. (2022)** [30]	Italy	14,000 ambulatory non-stress CTGs	Hybrid NN	Acc: 80.1%, AUC 0.81; Sens 69%; Spec 92%;	Enables earlier prediction of foetal compromise.
**Boudet et al. (2022)** [31]	France	635 CTGs	FSDop model (GRU with data augmentation and time delay correction)	Sens: 93.1%, PPV: 95.6%, Acc: 99.68%, AUC: 0.9992.	Improves detection of false maternal heart rate signals in CTG; enhances preprocessing for foetal monitoring and DL-based foetal distress detection.
**Frasch et al. (2021)** [32]	USA	36 CTGs	SSD (Single Shot MultiBox Detector)	Acc: 93.6%; Prec: 87%; Rec: 82.5%	Enables early detection of foetal compromise.
**Fotiadou et al. (2021)** [33]	Netherlands	Private dataset: 28 CTGs Public: 68 CTGs	Ensemble of CNN-LSTM with HR reliability classifier	Private test set: MAE = 2.0 bpm, MSE = 49.4 bpm^2^, PPA = 97.3%, Coverage = 87.9% PhysioNet: MAE = 1.1 bpm, MSE = 6.9 bpm^2^, PPA = 99.6%, Coverage = 82%.	Improves monitoring robustness by identifying unreliable segments
**Liu LC et al. (2021)** [34]	Taiwan	323,922-min CTGs (2605 for training/validation; 634 for testing)	Modified FCN	AUC 0.892; κ 0.525; sensitivity 0.528; FPR 0.632	Enables early detection of foetal compromise.
**Signorini MG et al. (2020)** [35]	Italy	120 CTGs	RF	AUC 0.974; Sens 0.891; Spec 0.870; PPV 0.891; NPV 0.899	Provides an interpretable, early antenatal IUGR screening tool from routine CTG.
**Hoodbhoy et al. (2019)** [36]	Pakistan	2126 CTGs	XGBoost	XGBoost: Prec >92% for pathological class; high precision (>96%) for suspect and pathological in training data	Useful for identifying high-risk pregnancies in low-resource settings.
**Zhao Z et al. (2019)** [37]	China & Turkey	552 CTGs	8-layer CNN using RP images	Acc: 98.69%, Sens: 99.29%, Spec: 98.10%, AUC: 98.70%	DL model for automated foetal hypoxia prediction in clinical settings
**Cömert et al. (2019)** [38]	Turkey	552 CTGs	SVM with a reduced feature set of 12 relevant features	Sens: 77.40%, Spec: 93.86%	Potential of combining feature selection algorithms with ML models to improve the prediction of foetal hypoxia.
**Zhao Z et al. (2019)** [39]	China	552 CTGs	2D CNN with 5×5 kernel, 15 filters, image resolution 64×64	Acc: 98.34%, Sens: 98.22%, Spec: 94.87%, Quality Index: 96.53%, AUC: 97.82%	Enables early detection of foetal compromise.
**Tang H et al. (2018)** [40]	China	24,360 twenty-minute FHR time-series samples	MKNet (CNN)	MKNet: Acc 94.7%; AUC 0.95; MKRNN: Acc 90.3%; AUC 0.91	Real-time automated FHR interpretation on portable devices.
**Leonarduzzi R et al. (2015)** [41]	France	3049 CTG s	Sparse SVM with *p* = 0.25	AUC: 0.71; Sens: 0.70; Spec: 0.70	Improves foetal acidosis detection during labour via advanced signal complexity analysis.
**Maeda K et al. (2010)** [42]	France	29 CTGS	ANN	Acc: 86% (internal test on 29 cases); Sens, Spec, PPV, NPV: all 100% for neural index	Provides a fully numeric, objective FHR analysis framework.
**Salamalekis E et al. (2002)** [43]	Greece	61 CTGs	Self-Organising Map neural network	Sens 83.3%; Spec 97.9% for identifying acidemic fetuses (umbilical pH < 7.20)	Enables early detection of foetal compromise.
**Liszka-Hackzell JJ. et al. (2001)** [44]	Sweden	34 CTGS for training; 38 CTGs for testing	Hybrid SOM-BP model using CTG-derived feature vectors	High accuracy	Early demonstration of AI use in CTG pattern recognition.
**Kol S et al. (1995)** [45]	Israel	Nonstress test records	ANN	Sens: 88.9%; FPR: 4.3%	Evaluates ANN for nonstress tests.
**Keith RD et al. (1994)** [46]	United Kingdom	50,000 five-minute CTG segments	Back-propagation NN on deceleration subtask	NN_5_ agreement with experts: ~75% vs. System 8000: ~47%; convergence in ~24 h for deceleration magnitude classification	Automated feature extraction to support an expert-system for real-time labour decision support

**Table 2:** AI in prediction of preterm delivery.

Reference	Country	Data Source	Best-Performing AI Model	Performance Metrics	Predictors	Clinical Utility
**Kloska A et al. (2025)** [47]	Poland	28 preterm, 22 term deliveries	Boosted Linear SVM	Acc 82%, Precision 83%, Recall 86%, F1-score 84%	CBC (WBC, PLT, Hb, HCT), CRP, BMI, parity, gestational diabetes, education level, etc.	Early detection of PTB using low-cost and routinely collected clinical data
**Ohtaka A et al. (2024)** [48]	Japan	30 preterm, 29 term deliveries	Xception CNN	Acc 0.718, AUC 0.704. VGG16: acc 0.654, Recall 0.808	Segmented transvaginal ultrasound images of the cervix at admission	Image-based prediction of PTB in high-risk pregnancies
**Bitar G et al. (2024)** [49]	USA	12,440 deliveries	XGBoost	Derivation cohort AUC: 0.70; Validation cohort AUC: 0.63	Multiple gestation, number of emergency department visits in the year prior to the index pregnancy, initial body mass index, gravidity, prior preterm delivery	Early detection of preterm birth using low-cost and routinely collected clinical data
**Ushida T et al. (2023)** [50]	China	31,157 infants <32 weeks GA and ≤1500 g	GBDT	AUROC: In-hospital death: 0.855; Short-term adverse outcomes: 0.750; Medium-term adverse outcomes: 0.701	12 antenatal variables: maternal age, gestational age, parity, delivery mode, diabetes, HDP, chorioamnionitis, PROM, ACS, foetal sex, birth weight, chorionicity	Improved predictive accuracy for mortality and neurological outcomes in extremely preterm infants using only antenatal variables.
**Andrade Júnior VL et al. (2023)** [51]	Brazil	524 singleton pregnancies (18–24 weeks)	SBELM (NN stacking)	At 10% FPR: AUC 0.808, Sens 47.3%, Spec 92.8%, PPV 32.7%, NPV 96.0%.	Cervical funneling, cervical length, index (CL/internal angle), previous PTB < 37 w, previous curettage, weight ≤ 58 kg, non-smoker status, absence of antibiotics use	Viable clinical tool for sPTB < 35 w screening during 2nd-trimester
**Kokkinidis I et al. (2023)** [52]	Greece	375 pregnant women (128 PTB)	Voting ensemble (XGBoost, RF, MLP)	AUC: 0.84, acc: 81%; F1-score: 0.70	32 features: demographics, social history, obstetric history, and clinical screening variables	Early detection of preterm birth using low-cost and routinely collected clinical data
**Khan W et al. (2023)** [53]	United Arab Emirates	3509 (deliveries	XGBoost	AUC 0.735 (parous), 0.723 (nulliparous)	35 selected features including: prior PTB, caesarean history, pre-eclampsia, BMI at delivery, maternal age, placenta previa, amniotic infection, physical activity, smoking	Personalised PTB risk interpretation for parous and nulliparous women.
**Zhang Y et al. (2023)** [54]	China	5411 deliveries	AdaBoost	Acc 0.954, Recall 0.985, Precision 0.963, F1-score 0.969, AUC 0.93.	21 EHR-derived features including parity, placenta previa, PPROM, diabetes, multiple gestation, etc.	Early detection of preterm birth using low-cost and routinely collected clinical data
**Sun Q et al. (2022)** [55]	China	9550 deliveries (4775 PTB, 4775 controls)	RF	Acc 0.816, AUC 0.891 (95% CI: 0.871–0.901), Sens 0.751, Spec 0.882	Age, magnesium, fundal height, MPV, waist size, total cholesterol, triglycerides, WBC count, and several others from blood/urine/physical exams	Early detection of preterm birth using low-cost and routinely collected clinical data
**Wong K et al. (2022)** [56]	Australia	≈ 953,000 births, 8.6% PTB	MLP	At 5% FPR (90% spec): MLP AUC 86.43%, F1 50.44%, Sens 52.69%, PPV ≈ 48%	Maternal socio-demographics, chronic conditions, pregnancy complications, past obstetric history, family history	Early detection of preterm birth using low-cost and routinely collected clinical data
**Zhou Y et al. (2022)** [57]	China	65,565 deliveries	GAM	U-shaped FT4–PTB association (*p* < 0.001); low FT4: HR 1.34 (95% CI 1.13–1.59); high FT4: HR 1.41 (95% CI 1.13–1.76)	First-trimester maternal FT4	Enables early risk stratification of PTB based on non-linear FT4 associations to inform surveillance and intervention planning.
**Park S et al. (2022)** [58]	South Korea	94 deliveries (38 PTB, 56 term deliveries)	SVM with bacterial risk scores and white blood cell (WBC) data	Sens: 71% (bacterial risk score only), 77% (with WBC data). Spec: 59% (bacterial risk score only), 67% (with WBC data)	Bacterial risk scores from cervicovaginal fluid, focusing on the ratios of *Lactobacillus iners* and *Ureaplasma parvum*	Potential for non-invasive prediction of PTB using cervicovaginal fluid bacterial profiles.
**Rawashdeh H et al. (2020)** [59]	Australia	274 cervical cerclage cases	RF (both classification and regression)	Classification task (delivery before 26 weeks): acc 95%, Sens 100%, G-mean 0.96, AUC 0.98.	Maternal age, parity, previous PTB/miscarriages, cervical length, cervical status, progesterone use, symptoms, multiple gestation, uterine anomalies, indication/type of cerclage	(1) Pre-cerclage counseling tool for PTB risk before 26 weeks, (2) Timeline prediction for delivery to optimise neonatal ICU preparedness and care planning.
**Gao C et al. (2019)** [60]	USA	25,689 deliveries	Ensemble of LSTM-WORD2VEC models (trained on 30 balanced datasets)	AUC 0.827, Sens 0.965, Spec 0.698, PPV 0.033.	Temporal EHR medical concepts (diagnoses, procedures, meds, labs) before 20 weeks GA	Early detection of preterm birth using low-cost and routinely collected clinical data
**Elaveyini U et al. (2011)** [61]	India	50 women with first trimester bleeding	ANN with 7 input neurons	acc: 70%	Maternal age, gestational age at bleeding, duration, amount, episodes of bleeding, presence of hematoma, placental location	PTB risk stratification in pregnancies with first trimester bleeding.
**Catley C et al. (2006)** [62]	Canada	~48,000 deliveries, verified on 19,710 deliveries	ANN with two hidden layers and weight elimination technique	Sens: 54.8%, Spec: 85.1–92.9%, AUC up to 0.73	8 obstetrical variables: maternal age, parity, previous term births, previous PTBs, multiple gestation, fetus’s gender, intention to breastfeed, smoking after 20 weeks	Early detection of preterm birth using low-cost and routinely collected clinical data
**Goodwin LK et al. (2001)** [63]	USA	19, 970 deliveries	Custom classifier (statistical + case-based + CART hybrid)	Custom classifier on femographic only (7 variables): AUC 0.72; All variables: AUC 0.75	Maternal age and binary coding for county of residence, education, marital status, payer source, race, and religion demographic characteristics	Early detection of preterm birth using low-cost and routinely collected clinical data
**Woolery LK et al. (1994)** [64]	USA	18,890 deliveries	LERS (Rough Set Theory-based Rule Induction)	Acc (expert system using LERS rules): Database 1: 88.8% Database 2: 59.2%, Database 3: 53.4%	214 variables including demographics, high-risk factors; medical and intervention history, ICD-9 codes	Early detection of preterm birth using low-cost and routinely collected clinical data

**Table 3:** AI in pre-eclampsia.

Reference	Country	Data Source	Best-Performing AI Model	Performance Metrics	Predictors	Clinical Utility
**Zheng W. et al. (2025)** [65]	China	Sagittal T2-weighted placental MRI from 420 pregnancies (140 PE, 280 normotensive)	LR on fused radiomic + DL features	Dice (segmentation): 0.917; AUC (PE vs. normotensive): train 0.839, test 0.858, internal val 0.888, external val 0.843	Radiomic wavelet, shape, texture features; five deep-learning components	Automated placental MRI analysis to identify PE and stratify FGR risk.
**Wang Z et al. (2025)** [66]	China	GEO microarrays cohorts	RF	AUC 0.792 (test)	11 IRDEGs (ADIPOR2, CD72, DDX17, FGF11, LCN6, NEDD4, NR1D1, NR2C1, RXRG, TMSB4X, VEGFA)	Blood-based 11-gene panel for early PE prediction and insight into immune dysregulation mechanisms
**Liu X et al. (2025)** [67]	China	GEO microarray cohorts	XGBoost	AUC 0.792	CRKL; STK31; HTRA4; EPHB3; PAPPA2	Potential diagnostic biomarkers and targets for PE.
**Lv B et al. (2025)** [68]	China	EHR data from 1040 women (PE incidence 6.8%)	XGBoost	Training AUC 0.963, F1 0.554; Test AUC 0.936, F1 0.488	Pre-pregnancy BMI; pregnancies count; MAP; smoking; AFP MoM; conception method	Early PE risk stratification using routine antenatal data to guide aspirin prophylaxis and monitoring.
**da Silva SMS et al. (2025)** [69]	Brazil	30 pregnant women (15 PE, 15 controls) and 30 matched newborn samples	PLS-DA	Newborn vs. pregnant: 99.7% acc using 10 wavenumbers; PE vs. control (newborn): ≤63% acc even with 100 features; maternal PE vs. control: <55% accuracy	Wavenumbers corresponding to carotenoids, DNA/RNA (PO_2_^−^), collagen/proteins, lipids/fatty acids	Demonstrates feasibility of screening for hypertensive pregnancy via plasma Fourier-transform infrared (FT-IR) spectroscopy.
**Eberhard BW et al. (2024)** [70]	USA	EHR data from 66,425 deliveries	Modified DeepHit deep survival NN	Time-dependent concordance index (Ctd): 0.839; Time-dependent AUC: 0.824; Overall survival AUC: 0.778	Age; race/ethnicity; chronic hypertension; parity; SBP/DBP; heart rate; platelets; creatinine; engineered temporal features up to 20 weeks’ gestation	Early PE risk stratification using routine antenatal data to guide aspirin prophylaxis and monitoring
**Zhou T. et al. (2024)** [71]	China	Retinal fundus photographs obtained before 20 weeks’ gestation in 1138 singleton pregnancies	Inception-ResNet-v2 CNN	AUC 0.883, Sens 0.722, Spec 0.934	Retinal vascular features encoded in fundus score (reflecting microvascular changes), plus maternal age, BMI, parity, chronic hypertension, prepregnancy BMI category	Early PE risk stratification using routine antenatal data to guide aspirin prophylaxis and monitoring
**Vasilache I-A et al. (2024)** [72]	Romania	EHR data from 210 singleton pregnancies	RF	PE acc 96.3%; IUGR 95.9%; early IUGR 96.2%; late IUGR 95.2%; PE + IUGR association 95.1% (sens/spec ≥ 90%)	Maternal age; BMI; nulliparity; conception type; smoking; history of PE/IUGR/preterm birth/autoimmune/CKD/DM/HTN; MAP; β-HCG, PAPP-A, PlGF, PP-13 (all MoM)	Early PE risk stratification using routine antenatal data to guide aspirin prophylaxis and monitoring
**Bülez A et al. (2024).** [73]	Turkey	HER data from 10,307 women (1158 PE, 9194 controls)	LightGBM	Sens 73.7%; Spec 92.7%; Acc 90.6%; AUC 0.832	Hemoglobin; age; AST; ALT; blood group; plus sociodemographics, vitals	Early PE risk stratification using routine antenatal data to guide aspirin prophylaxis and monitoring
**Kaya Y. et al. (2024)** [74]	Turkey	EHR data from 100 women admitted in 1st trimester	XGBoost	Acc 70% (nulliparous), 72.7% (parous); AUC-ROC 0.64/0.767; Sens 80%/60%; Spec 60%/83.3%	Maternal age; BMI; smoking; history of DM, GDM, HTN, SLE-APS; gravida; parity; MAP; previous PE	Early PE risk stratification using routine antenatal data to guide aspirin prophylaxis and monitoring
**Tiruneh et al. (2024)** [75]	Australia	EHR data from 48,250 women	RF	AUC 0.84, acc 0.79	Maternal age; ethnicity; BMI; parity; prior PE history; nulliparity; history of GDM; pre-existing hypertension; diabetes; family history of hypertension/diabetes/PE; renal disease; smoking; PCOS	Early PE risk stratification using routine antenatal data to guide aspirin prophylaxis and monitoring
**Huang P et al. (2024)** [76]	China	GEO datasets (80 PE, 77 controls); validation cohort (12 PE, 12 controls)	LR	AUC (3-gene model): 0.871; individual genes AUCs > 0.70	CPOX, DEGS1, SH3BP5 gene expression	Provides a 3-gene blood-based diagnostic signature enabling early, noninvasive PE detection.
**Araújo DC et al. (2024)** [77]	Brazil	EHR data from 132 women (65 severe PE, 67 controls)	LightGBM	AUROC 0.90 ± 0.10; Sens 0.95; Spec 0.79; Acc 0.87; Precision 0.82	Neutrophils, mean corpuscular hemoglobin (MCH), aggregate index of systemic inflammation (AISI)	Supports third-trimester sPE diagnosis using routine CBC.
**Li T et al. (2024)** [78]	nan	EHR data from 4644 pregnancies (49 preterm PE, 161 term PE cases)	Voting Classifie	All PE: AUC 0.831; DR_10_ 0.513 Preterm PE: AUC 0.884; DR_10_ 0.625	Maternal age, height, pre-pregnancy weight, parity, conception method, history of PE/HTN/CKD/DM; MAP; UtA-PI; PAPP-A; PlGF	Early PE risk stratification using routine antenatal data to guide aspirin prophylaxis and monitoring
**Gil MM et al. (2024)** [79]	Spain	EHR data from 10,110 1st trimester pregnancies	NN	Early PE DR 84.4%; AUC 0.920; Preterm PE DR 77.8%, AUC 0.913; All PE DR 55.7% (49.0–62.2), AUC 0.846	Maternal factors, MAP, UtA-PI, PlGF	Enables non-MoM–based first-trimester screening for PE.
**Edvinsson C. et al. (2024)** [80]	Sweeden	EHR data from 81 women (41 severe PE, 40 controls)	XGBoost	Test acc 0.82, AUC 0.85; Cross-val acc 0.88, AUC 0.91.	AST, uric acid, BMI	Early PE risk stratification using routine antenatal data to guide aspirin prophylaxis and monitoring
**Ansbacher-Feldman Z. et al. (2022)** [81]	UK	EHR data from 60,789 1st trimester pregnancies	NN	PE: AUC 0.82; Preterm PE: AUC 0.91	Maternal age, BMI, parity, prior PE, interpregnancy interval, race/ethnicity, IVF status; MAP; UtA-PI; PlGF; PAPP-A	Early PE risk stratification using routine antenatal data to guide aspirin prophylaxis and monitoring
**Villalaín C. et al. (2022)** [82]	Spain	EHR data from 215 singleton early-onset PE cases	SVM	AUC 0.79; sens 77.3%; spec 80.1%; PPV 81.5%; NPV 76.2%	Age; BMI; prior PE; gestational age; SBP/DBP; platelets; creatinine; AST/ALT; sFlt-1, PlGF; Doppler indices; foetal biometry	Provides individualized risk of imminent delivery and severe complications in PE.
**Liu M et al. (2022)** [83]	China	EHR data from 11,152 pregnancies	RF	AUROC 0.86 (95% CI 0.80–0.92), acc 0.74, precision 0.82, recall 0.42, F1 0.56; Brier score 0.17, calibration slope 0.92, intercept 0.20	Age; BMI; weight; height; GA; parity; chronic HTN; prior DM; prior PE; MAP; free β-hCG; PAPP-A; uterine artery PI	Early PE risk stratification using routine antenatal data to guide aspirin prophylaxis and monitoring
**Bennett R et al. (2022)** [84]	USA	Texas units (360,943 deliveries, 3.98% PE), Oklahoma units (84,632 deliveries, 5.58% PE), and MOMI cohort (31,431 deliveries, 8.73% PE)	Cost-sensitive DNN with focal loss & weighted cross-entropy	AUC Texas: 0.66; AUC Oklahoma: 0.64; AUC External (MOMI): 0.77	Demographics, comorbidities, prenatal labs, BMI, BP spikes, and temporal features (varies by dataset)	Early PE risk stratification using routine antenatal data to guide aspirin prophylaxis and monitoring
**Hoffman MK et al. (2021)** [85]	USA	EHR data from 20,032 pregnancies	NN	At a 10% FPR: detects 53% of all PE cases (vs 41% without biomarkers) and 75% of preterm PE (vs 53% without)	Age, BMI, parity, prior PE, MAP, UtA-PI, PlGF, PAPP-A	Enables non-MoM–based first-trimester screening for overall and preterm PE.
**Wang G. et al. (2021)** [86]	China	EHR from 907 women with PE	RF	AUC 0.711 (95% CI 0.697–0.726); acc 0.817; sens 0.815; spec 0.984; PPV 0.777; NPV 0.807	SBP, BUN, neutrophil count, glucose, D-Dimer (top five of 20 clinical and lab features)	Identifies high-risk women for targeted CVD prevention and monitoring after PE.
**Sufriyana H et al. (2020)** [87]	Indonesia	EHR data 3318 PE/eclampsia, 19,883 controls	RF	AUROC (external validation): geographical split 0.88; temporal split 0.86 (95% CI 0.85–0.86)	17 features from demographics and medical history over 24 months (e.g., age, parity, comorbidities, prior hospitalizations)	Early PE risk stratification using routine antenatal data to guide aspirin prophylaxis and monitoring
**Jhee JH et al. (2019)** [88]	South Korea	EHR data from 11,006 pregnancies	SGB	AUC 0.924; acc 0.973	SBP; DBP; BUN; creatinine; platelet count; WBC; calcium; UPCR; demographics, medical history, labs pattern-cluster features	Enables early prediction of late-onset PE.

**Table 4:** AI in gestational diabetes.

Reference	Country	Data Source	Best- Performing AI Model	Performance Metrics	Predictors	Clinical Utility
**Bigdeli SK et al. (2025)** [89]	Iran	EHR data from 16,730 pregnancies	RF	Insulin model: AUC 0.64; acc 0.62; precision 0.60; recall 0.63; GTT model: AUC 0.94; acc 0.89; precision 0.86; recall 0.92	Demographics; medical history; clinical findings; first-trimester FBS, Hb, Hct, Cr, PLT, vit D3, NT sonographic markers	First-trimester GDM risk stratification
**Zhao M. et al. (2025)** [90]	China	EHR data from 103,172 pregnancies (15,138 GDM; 88,034 controls)	MLP with NearMiss	AUC 0.943; acc 0.884	BMI; age; age of menarche; higher education; folic acid supplementation; family history of DM; HGB; WBC; PLT; Scr; HBsAg; ALT; ALB; TBIL	First-trimester GDM risk stratification
**Zaky H et al. (2025)** [91]	Qatar	EHR data from 138 pregnancies (63 GDM, 75 controls)	Stacking ensemble	Acc 88.8%; recall 92.1%; precision 87.3%; F1-score 89.6%	History of high glucose/diabetes, HbA1c%, glucose, insulin, NT-proBNP, lipids, electrolytes, blood counts, liver/renal markers, hormones, family history, vitamins	First-trimester GDM risk stratification
**Zhou H et al. (2025)** [92]	China	2D ultrasound images at 11–13 weeks: discovery (*n* = 305; 139 GDM, 166 controls) and independent validation (*n* = 110; 53 GDM, 57 controls)	Nomogram (radiomics + DLCNN + clinical)	Discovery AUC 0.93, Validation AUC 0.88	Radiomics features; age; pre-pregnancy BMI; DLCNN score	First-trimester GDM risk stratification
**Chen M et** **al. (2024)** [93]	China	EHR data from 588 women with two consecutive singleton deliveries and index-pregnancy GDM	LGB	AUROC 0.942	First-trimester FPG, 1–2 h OGTT glucose, triglycerides, cholesterol, HbA1c, macrosomia, preterm birth, age > 35 y, abdominal circumference, gestational weight gain	First-trimester GDM risk stratification
**Kaya et al. (2024)** [94]	Turkey	EHR data from 97 pregnancies	XGB Classifier	Acc 66.7%, AUC 0.55; sens 80%, spec 50%	Age; BMI; gravida; parity; previous birth weight; smoking; first-visit plasma glucose; family history of DM	First-trimester GDM risk stratification
**Cubillos G. et al. (2023)** [95]	Chile	EHR data from 1611 pregnancies	MLP with optimised hyperparameters	Sens 0.82; Spec 0.72–0.74; Acc 0.73–0.75; AUCROC 0.81	First-trimester fasting glycemia, age, BMI, weight, gravidity	First-trimester GDM risk stratification
**Hu X et al. (2023)** [96]	China	EHR data from 735 pregnancies (training set) and 190 pregnancies (testing set)	XGBoost	AUC 0.946; acc 0.875	20 first-trimester variables (e.g., previous GDM, age, HbA1c, MAP, lipids, liver enzymes)	First-trimester GDM risk stratification
**Houri O et al. (2023)** [97]	Israel	EHR data from 452 GDM pregnancies	NN	Acc: 82% at GDM diagnosis; 91% at delivery	Age; parity; gravidity; pre-pregnancy BMI; GCT; OGTT values; maternal weight (pre-preg, at diagnosis, at delivery); treatment type; glycemic control	First-trimester GDM risk stratification
**Kadambi et al. (2023)** [98]	USA	Monitoring Mothers-to-be (nuMoM2b) EHR data	LR	AUC 0.74	Maternal race; BMI at first visit; prepregnancy BMI; family history of GDM; hypertension; valvular heart disease; structural heart disease; coronary artery disease; cardiac arrhythmia; polycystic ovary syndrome	First-trimester GDM risk stratification
**Watanabe M. et al. (2023)** [99]	Japan	EHR data from 82,698 GDM pregnancies	GBDT	AUC 0.67 for recurrent GDM; AUC 0.74 for new-onset GDM	775 variables covering pre-pregnancy lifestyle, anthropometrics, smoking, diet, SF-8 QOL, K6 distress, lab values, etc.	First-trimester GDM risk stratification
**Zhou M et al. (2022)** [100]	China	492 GDM pregnancies with 2D ultrasound scans within 3 days before delivery	ANN	MAE 153.5 g; MAPE 4.7%; ANN vs. Hadlock: MAE 148.5 g vs. 192.2 g (*p* < 0.001)	Foetal biometry from ultrasound; maternal anthropometrics	Enhances foetal weight estimation accuracy in GDM
**Kumar M. et al. (2022)** [101]	Singapore	S-PRESTO cohort (*n* = 222)	Gradient boosting classifier + linear SVM	AUC 0.93	HbA1c, mean BP, fasting insulin, triglycerides/HDL ratio	Preconception risk stratification for GDM; deployable via web app
**Kumar M. et al. (2022)** [102]	Singapore	GUSTO mother-offspring cohort (*n* = 909)	CatBoost	AUC 0.82	mean arterial BP at booking; maternal age; previous history of GDM; ethnicity (Chinese/Indian vs. Malay)	First-trimester GDM risk stratification deployable via web app
**Yang J. et al. (2022)** [103]	UK	OUH GDm-Health system: 1148 GDM pregnancies; external validation: 709 cases.	XGBoost regression	Internal (OUH): MSE 0.021, R^2^ 0.482, MAE 0.112; External (RBH): MSE 0.020, R^2^ 0.519, MAE 0.108	Pre-/post-breakfast, post-lunch, post-dinner glucose readings; engineered “High-Readings” and “Gradients”; maternal age; gestational day; medication status	Predicts short-term hyperglycemia risk to guide timely clinical monitoring and intervention.
**Liao LD et al. (2022)** [104]	USA	EHR data from 30,474 GDM pregnancies: discovery (*n* = 27,240) and validation (*n* = 3234)	Super learner (LASSO, CART, RF, XGBoost)	AUC 0.934 (discovery)/0.815 (validation)	Demographics, clinical history, OGTT/glucose challenge values, SMBG metrics, labs across four timepoints	Early triage for pharmacologic treatment of GDM
**Du Y. et al. (2022)** [105]	Ireland	EHR data from 484 overweight/obese women	SVM	AUC-ROC 0.792; AUC-PR 0.485; balanced ACC 0.751	Family history DM; weight; WBC; fasting glucose; insulin	First-trimester GDM risk stratification in overweight/obese women.
**Araya J. et al. (2021)** [106]	Chile	EHR data from 39 pregnancies (33 NGT, 6 GDM)	Principal component analysis	Spontaneous clustering of GDM vs. NGT	FT4, TT3, TT4, TSH (1st & 2nd trimester); OGTT; diastolic blood pressure; prior GDM	Suggests thyroid hormone profiling may augment early GDM diagnosis beyond OGTT.
**Liu H et al. (2020)** [107]	China	EHR data from 19,331 pregnancies	XGBoost	AUC 0.742	Fasting plasma glucose; pre-pregnancy BMI; alanine aminotransferase; maternal age; waist circumference; weight gain; family history of diabetes	First-trimester GDM risk stratification

**Table 5:** AI in postpartum haemorrhage.

Reference	Country	Data Source	Best-Performing AI Model	Performance Metrics	Predictors	Clinical Utility
**Ahmadzia HK et al. (2024)** [108]	USA	228,438 deliveries	Gradient Boosting	ROC-AUC 0.833; PR-AUC 0.210	50 antepartum and intrapartum characteristics and hospital characteristics; top features: mode of delivery; oxytocin incremental dose for labour; intrapartum tocolytic use; presence of anaesthesia nurse; hospital type	Identification of high-risk PPH parturients to guide proactive interventions
**Wang M et al. (2024)** [109]	China	6144 caesarean deliveries	RF	MAE 21.7 mL (< 5.4% error); RMSE 33.75 mL (< 9.3% error) on test set.	27 indicators: haemoglobin; WBC; platelets; PT; INR; APTT; TT; fibrinogen; Na; K; Cl; Ca; bilirubin; urea; creatinine; weight; height; infant weight; age; number of pregnancies; gestational week; blood pressures; complications; anaesthesia method; ASA class; emergency status; pregnancy days	Identification of high-risk PPH parturients during cesarian to guide proactive interventions
**Holcroft S. et al. (2024)** [110]	Rwanda	430 deliveries (108 PPH cases, 322 controls)	RF	Sens 80.7%, spec 71.3%, misclassification rate 12.19%	Haemoglobin level at labour; maternal age; no medical insurance; multiple foetuses; pre-labour bleeding; intrauterine foetal death; BMI; multiparity; history of PPH	Identifies women at high risk of PPH upon admission for targeted interventions
**Westcott JM et al. (2022)** [111]	USA	30,867 deliveries	GBDT	AUROC: 0.979, Acc: 98.1%, Sens: 76.3%	497 variables including demographics, obstetric/medical/surgical/family history, vital signs, lab results, labour medication exposures, and delivery outcomes	Identification of high-risk PPH parturients to guide proactive interventions
**Liu J et al. (2022)** [112]	China	10,520 vaginal deliveries	LGB + LR	AUC 0.803, Brier 0.061, F-measure 0.845, Sens 0.694, Spec 0.800	49 clinical variables (16 known high-risk factors + TOCO features such as contraction frequency, Mean_Area intensity; haematocrit; shock index; WBC; gestational hypertension; neonatal weight; second stage labour time; amniotic fluid volume; BMI; etc.)	Identification of high-risk PPH parturients after vaginal delivery
**Akazawa M et al. (2021)** [113]	Japan	9894 vaginal deliveries (188 PPH cases)	LR	AUC 0.708; Acc 0.686; FPR 0.312; FNR 0.398	11 clinical variables: age; parity; maternal height; weight before pregnancy; weight on admission; gestational age; birthweight; baby sex; foetal position; oxytocin use; delivery mode	Identification of high-risk PPH parturients during vaginal delivery to guide proactive interventions
**Venkatesh KK et al. (2020)** [114]	USA	152,279 deliveries	XGBoost	AUC≈0.93	55 maternal risk factors available at labour admission (from literature and expert consensus) were included—e.g., maternal demographics (age, race), obstetric history/diagnoses (placenta previa, foetal macrosomia, pre-eclampsia), comorbidities (chronic hypertension, diabetes), and initial vital signs	Identification of high-risk PPH parturients to guide proactive interventions

**Table 6:** AI in labour and delivery outcomes.

Reference	Country	Data Source	Best-Performing AI Model	Performance Metrics	Predictors	Clinical Utility
**Borycka K et al. (2025)** [115]	Czech Republic, Slovakia, Poland, Spain	Impedance spectroscopy and 3-D EAUS data from 152 deliveries	Ensemble tree-based ML model with 10-fold cross-validation	Overall acc 0.86; sens 0.67–0.95; spec 0.80–0.98	Impedance-derived spectral features; age; BMI; parity; head circumference; mode of delivery; time since delivery	Non-invasive, bedside detection of OASI to guide early intervention and repair decisions
**Hu T et al. (2025)** [116]	China	EHR data from 1191 vaginal deliveries (300 episiotomies)	SVM	SVM: Acc 0.793; Recall 0.981; Precision 0.790; F1 0.875; AUC 0.882.	Age; gestational age; parity; history of stillbirth; BMI; pregnancy complications; perineal length, elasticity, thickness, edema and skin tear; UC; duration of labour; shoulder dystocia; assisted breech; instrumental delivery; EFW; late deceleration; severe variable deceleration; amniotic fluid contamination; abnormal foetal position; working years of midwife; professional title; maternal cooperation	Decision support by predicting the risk of mediolateral episiotomy.
**Boie S et al. (2024)** [117]	Denmark and Netherlands	EHR data from 1198 deliveries	XGBoost	AUROC:0.75, AUPRC: 0.39	Maternal age, BMI, parity, cervical dilation, foetal station, oxytocin dosage, etc.	Individual risk assessment for caesarean delivery after active labour onset.
**Wong Ms et al. (2024)** [118]	USA	EHR data from 37,932 deliveries	Ensemble model chosen via AutoML	AUC: 0.82	Intrapartum clinical data (e.g., cervical dilation, FHR, uterine activity)	Supports dynamic prediction of mode of delivery during labour using real-time data.
**Kuanara et al. (2024)** [119]	India	EHR data from 101 deliveries	DNN	Train: AUC 0.99; KS 0.98; error rate caesarean 0.02, vaginal 0.00; Test: error rate caesarean 0.20, vaginal 0.10	Mother’s weight, height, age, GA, Hb, FHF amniotic fluid index, cervix length, child birth weight, pregnancy count	Clinical decision support in selecting mode of delivery.
**Xu J et al. (2024)** [120]	China	EHR data from 100 deliveries in training set, 50 in validation set	GNB	Training AUC: 0.82, Validation AUC: 0.79, Acc: 80.9%, Sens: 72.7%, Spec: 75.0%, Precision: 84.2% F1 Score: 0.78	Angle of progression, cervical length, subpubic arch angle, estimated foetal weight	May assist in early prediction of spontaneous vaginal delivery failure in term nulliparous women.
**Chen G et al. (2024)** [121]	China and New Zealand	Retrospective image collection from 1124 parturients	UNet variants	Dice Coefficients (Segmentation Accuracy): 89.04–90.02%	Segmentation targets include pubic symphysis and foetal head from transperineal ultrasound images; used to compute angle of progression	Enables development of AI tools for objective, automated assessment of foetal head descent and prediction of delivery mode.
**Liu Ys et al. (2023)** [122]	China	EHR data from 101 deliveries	XGBoost	MAE 13.49 h, RMSE 16.98 h	Age, BMI, gestational age, cervical length, foetal weight, BPD, Bishop score components, etc.	Potential to improve prediction of labour induction outcomes over traditional Bishop score
**Lodi et al. (2023)** [123]	France	EHR data from 410 class III obese nulliparous women with attempted vaginal delivery	Probability Forest	AUC: 0.70, Acc: 0.66, Sens: 0.44, Spec: 0.87	Initial maternal weight, labour induction	Support personalised counseling on delivery mode in late pregnancy in class III obese nulliparous women.
**Zhang R et al. (2023)** [124]	China	EHR data from 2552 deliveries (training *n* = 2025; validation *n* = 527)	RF	Accuracy 0.8956; MCC 0.7530; AUC-ROC 0.9791; AUC-PRC 0.9579	Age; maternal height; weight at delivery; weight gain; parity; assisted reproduction; abnormal blood glucose; hypertensive disorders; scarred uterus; PROM; placenta previa; abnormal foetal position; thrombocytopenia; floating foetal head; labour analgesia	Predicts likelihood of caesarean section to support clinicians in individualized delivery planning.
**D’Souza et al. (2023)** [125]	Canada, UK, USA, Switzerland	EHR data from 1107 participants with singleton pregnancies and Bishop Score <4, undergoing induction of labour with dinoprostone vaginal insert	ML model not specified	AUROC: 0.73	Parity, gestational age (37–41 weeks), maternal BMI, maternal age, maternal comorbidities, Bishop score	Prediction of successful labour induction in women with a low Bishop score.
**Myer R et al. (2022)** [126]	Israel	EHR data from 73,667 deliveries (train: 48,084; validation: 12,016; test: 13,567)	XGBoost	XGBoost AUC: Training: 0.874, Validation: 0.839, Test: 0.840	13 features (e.g., maternal age, BMI, cervical dilation, effacement, labour onset, ultrasound-adjusted foetal biometry, parity)	Web calculator (BirthAI.org) to predict unplanned caesarean delivery for individualized counseling.
**Hu T et al. (2022)** [127]	China	EHR data from 907 participants (primipara *n* = 495; multipara *n* = 312)	LR	Primipara: AUC 0.84; acc 90.3%; recall 0.986; precision 0.908; F1 0.943. Multipara: AUC 0.89; acc 97.1%; recall 0.993; precision 0.977; F1 0.982.	Age; height; weight; BMI; gestational age; previous caesareans; number of abortions; Bishop score; foetal weight; amniotic fluid index; amniotic fluid contamination; foetal head circumference; foetal abdominal circumference; biparietal diameter; femur length; uterine height; abdominal circumference; membrane status; labour analgesia	Allows clinicians to estimate probability of successful oxytocin-induced labour at admission.
**Ghi T et al. (2022)** [128]	Europe, Asia, Africa	EHR data from 1219 term pregnancies in second stage of labour	Pattern-recognition feed-forward NN	Overall acc 90.4%; foetal occiput anterior (OA) acc 91.1%; non-OA acc 89.3%; F1-score 88.7%; PR-AUC 85.4%; Cohen’s κ = 0.81	Transabdominal and transperineal ultrasound (TPU) images parameters for detection of foetal position	Rapid, automatic classification of foetal OA vs. non-OA on TPU to aid in labour wards.
**Islam Ms et al. (2022)** [129]	Bangladesh and Saudi Arabia	Pakistan Demographic and Health Survey (PDHS) 2012–13 and 2017–18 datasets	HGSORF (Random Forest optimized with HGSO)	Acc: 98.33%, Sens: 98.33%, Spec: 98.33%, Precision: 98.34%, AUC: ~99%	24 features including: maternal age, BMI, ANC visits, previous C-section, household size, domestic violence, husband’s education and occupation, etc	High-potential decision support system (DSS) for predicting CS likelihood; includes XAI tools (SHAP and LIME) to improve interpretability
**Chill Hh et al. (2021)** [130]	Israel	EHR data from 98,463 deliveries (323 OASI cases)	CatBoost gradient boosting	AUC 0.756	Parity; number of previous births; maternal weight; GA; birth weight; head circumference; induction method; duration of second stage	Stratification of women by OASI risk.
**Ullah Z et al. (2021)** [131]	Saudi Arabia	EHR data from 80 deliveries	k-NN on enriched data	Acc: 84.38%	Age, delivery number, delivery time (premature, timely, latecomer), blood pressure status, FHR	Demonstrates potential of ML models to predict mode of delivery.
**Guedalia J et al. (2021)** [132]	Israel	EHR data from 73,868 term deliveries in second stage of labour	Gradient Boosting	AUC: 0.761; Sens: 72.1%, OR: 5.3 for high-risk vs. low-risk group	Antepartum features and intrapartum data gathered during the first stage of labour	Enables early identification of high-risk deliveries for severe adverse neonatal outcomes.
**Tarimo Cs et al. (2021)** [133]	Tanzania	EHR data from 21,578 deliveries	Boosting	Boosting model: AUC: 0.75, Acc: 0.74, Sens: 0.85, Spec: 0.59, PPV: 0.75, NPV: 0.73	Maternal age, parity, gestational age, BMI, birth weight, PROM, multiple gestation, maternal education, marital status, occupation, alcohol use	Provides insight into early identification of candidates for labour induction using routine data.
**Meyer R et al. (2020)** [134]	Israel	EHR data from 989 consecutive singleton TOLAC deliveries	RF	AUC-PR 0.351 ± 0.028,	Prior vaginal delivery, maternal height, prior arrest of descent, maternal weight, gestational age, etc.	Enhanced prediction of TOLAC success.
**Ricciardi C et al. (2020)** [135]	Italy	EHR and CTG recordings from 370 deliveries	RF	Acc 91.1%, Sens 90.0%, Spec 92.2%, Precision 92.1%, AUCROC 96.7%	17 features: gestational age, FHR metrics, (UCs, accelerations/decelerations, spectral power (LF/HF), entropy, Poincaré plot axes, etc.	Helps predict the type of delivery from intrapartum CTG signals
**Beksac Ms et al. (2018)** [136]	Turkey	HER data from 800 deliveries (600 vaginal births and 200 caesarean sections)	ANN with back-propagation	Sens: 60.9%, Spec: 97.5%, PPV: 81.8%, NPV: 93.1%, Test Efficiency: 91.8%	Maternal age, gravida, parity, gestational age, labour induction type, presentation, risk factors	Provides a supportive decision tool to predict delivery mode.
**Fergus P et al. (2018)** [137]	UK	CTG recordings from 506 vaginal and 46 caesarean deliveries	Ensemble model combining FLDA, RF, and SVM	Sens 87%, Spec 90%, AUC 96%, MSE 9%	13 FHR features: including STV, SampEn, DFA, RMS, FD, SD1, SD2, SDRatio, RBL, accelerations, decelerations, etc.	Provides a supportive decision tool to predict delivery mode based on FHR alone.
**Macones Ga et al. (2001)** [134]	USA	HER data from 400 women with prior caesarean delivery (100 failed TOLAC, 300 successful VBAC)	Multivariate LR	Sens 77%, Spec 65%, Acc 69%	Substance abuse, prior successful VBAC, cervical dilation at admission, need for labour augmentation	Enhanced prediction of TOLAC success.
**Devoe Ld et al. (1996)** [138]	USA	EHR data from 200 term pregnancies with spontaneous labour (159 for training, 41 for testing)	Feedforward NN	Correlation with actual duration: r = 0.88 (NN), Compared to partogram: r = 0.35.	UC, EFW, foetal position, station, gestational age, maternal parity, age, height, weight, membrane status, cervical dilatation	Provides more accurate prediction of first-stage labour duration.

## Abbreviations

The following abbreviations are used in this manuscript:

2D	Two-Dimensional
AIF	Amniotic Fluid Index
ANN	Artificial Neural Network
APTT	Activated Partial Thromboplastin Time
ASA	American Society of Anesthesiologists Physical Status
ASAT	Aspartate Aminotransferase
ACS	Antenatal Corticosteroids
AdaBoost	Adaptive Boosting
Acc	Accuracy
AISI	Aggregate Index of Systemic Inflammation
AI	Artificial Intelligence
AN	Artificial Neural Network
AUROC	Area Under the Receiver-Operating-Characteristic Curve
AUC	Area Under the Curve
AUC-PR	Area Under the Precision–Recall Curve
AUCfinal	Overall Survival AUC
AUCtd	Time-Dependent AUC
Bagging	Bootstrap Aggregating
BMI	Body Mass Index
BP	Blood Pressure
CBC	Complete Blood Count
CART	Classification and Regression Tree
CHAID	Chi-Square Automatic Interaction Detection
CI	Confidence Interval
CKD	Chronic Kidney Disease
CS	Caesarean Section
CSL	Consortium on Safe Labor
Cox PH	Cox Proportional Hazards Model
CNN	Convolutional Neural Network
Cr	Creatinine
CTG	Cardiotocography
DFA	Detrended Fluctuation Analysis
DBP	Diastolic Blood Pressure
DEGs	Differentially Expressed Genes
DL	Deep Learning
DLR	Deep Learning Radiomics
DLCNN	Deep Learning Convolutional Neural Network
DM	Diabetes Mellitus
DNN	Deep Neural Network
DTC	Decision Tree Classifier
DT	Decision Tree
DR	Detection Rate
DR_10_	Detection Rate at 10% FPR
DRF	Distributed Random Forest
EHR	Electronic Health Record
EFM	Electronic Fetal Monitoring
EMG	Electromyography
EMR	Electronic Medical Record
EFW	Estimated Fetal Weight
F1	F1-Score
FBS	Fasting Blood Sugar
FGR	Fetal Growth Restriction
FHR	Fetal Heart Rate
FPG	Fasting Plasma Glucose
FT4	Free Thyroxine
FT-IR	Fourier-Transform Infrared Spectroscopy
FPR	False-Positive Rate
GB	Gradient Boosting
GBM	Gradient Boosting Machine
GBDT	Gradient-Boosted Decision Trees
GBT	Gradient-Boosted Trees
GAM	Generalized Additive Model
GCT	Glucose Challenge Test
GDM	Gestational Diabetes Mellitus
GA	Gestational Age
GNB	Gaussian Naïve Bayes
GRU	Gated Recurrent Unit
GWG	Gestational Weight Gain
HDP	Hypertensive Disorders of Pregnancy
HGSORF	Henry Gas Solubility Optimization–Based Random Forest
HELLP	Hemolysis, Elevated Liver Enzymes, Low Platelets Syndrome
HGB	Hemoglobin
Hb	Hemoglobin
HbA1c	Glycated Hemoglobin
HTN	Hypertension
ICU	Intensive Care Unit
IBS	Integrated Brier Score
IUGR	Intrauterine Growth Restriction
IVF	In Vitro Fertilization
KNN	k-Nearest Neighbors
k-NN	k-nearest Neighbors
LDA	Linear Discriminant Analysis
LASSO	Least Absolute Shrinkage and Selection Operator
LFHF	Low and High-Frequency Spectral Power
LR	Logistic Regression
LGB	LightGBM
LERS	Learning from Examples Using Rough Sets
LSTM	Long Short-Term Memory
MARS	Multivariate Adaptive Regression Splines
MAE	Mean Absolute Error
MAP	Mean Arterial Pressure
MAPE	Mean Absolute Percentage Error
MCH	Mean Corpuscular Hemoglobin
MCC	Matthews Correlation Coefficient
ML	Machine Learning
MLP	Multilayer Perceptron
MoM	Multiples of the Median
MOMI	Multi-center Observational Maternal Initiative
MRI	Magnetic Resonance Imaging
NB	Naïve Bayes
NFH	Neonatal Fetal Hypoxia
NMF	Non-negative Matrix Factorization
NICHD	National Institute of Child Health and Human Development
NPV	Negative Predictive Value
OASI	Obstetric Anal Sphincter Injury
OGTT	Oral Glucose Tolerance Test
PAPP-A	Pregnancy-Associated Plasma Protein-A
PCA	Principal Component Analysis
PF	Probability Forest
PG	Plasma Glucose
PlGF	Placental Growth Factor
PP-13	Placental Protein-13
PPROM	Preterm Premature Rupture of Membranes
PROM	Premature Rupture of Membranes
PPV	Positive Predictive Value
PR-AUC	Precision-Recall Area Under the Curve
Pred	Predictors
PT	Prothrombin Time
PTB	Preterm Birth
RF	Random Forest
ReLU	Rectified Linear Unit
RBF	Radial Basis Function
ROC	Receiver-Operating-Characteristic
ROC-AUC	Receiver-Operating-Characteristic Area Under the Curve
RMS	Root-Mean-Square
RNN	Recurrent Neural Network
SBELM	Stacked Bayesian Extreme Learning Machine
SBP	Systolic Blood Pressure
Sens	Sensitivity
SHAP	SHapley Additive Explanations
SFM	State Flow Machine
SGB	Stochastic Gradient Boosting
SMOTE	Synthetic Minority Oversampling Technique
SOM	Self-Organizing Map
spec	Specificity
ST	Study Type
STV	Short-Term Variability
TBIL	Total Bilirubin
TG	Triglycerides
TOCO	Tocodynamometry
TGLCN	Trend-Guided Long Convolution Network
T2WI	T2-Weighted Imaging
TT	Thrombin Time
UC	Uterine Contraction
UtA-PI	Uterine Artery Pulsatility Index
U-Net	U-Shaped Convolutional Network
US	Ultrasound
VD	Vaginal Delivery
Vit D_3_	Vitamin D_3_
VIP	Variable Importance in Projection
WBC	White Blood Cell Count
WC	Waist Circumference
XGB	Extreme Gradient Boosting
XGBoost	eXtreme Gradient Boosting
